# Effect of group IV content on elastic and defect properties of NbTiZr refractory medium-entropy alloys

**DOI:** 10.1080/14686996.2026.2736330

**Published:** 2026-09-18

**Authors:** Tomohito Tsuru, Ivan Lobzenko, Shu Han, Haruyuki Inui

**Affiliations:** a Research Center for LWR Engineering, Japan Atomic Energy Agency, Ibaraki, Japan; b Department of Materials Science and Engineering, Kyoto University, Kyoto, Japan

**Keywords:** Refractory medium-entropy alloys, screw dislocation core, elastic anisotropy, chemical short-range order, first-principles calculations

## Abstract

Some refractory high- and medium-entropy alloys exhibit an exceptional combination of strength and ductility, yet the origin of this behavior remains unresolved. Here, first-principles calculations are used to investigate how group IV elements and the associated valence electron concentration (VEC) influence the defect energetics of body-centered cubic refractory alloys. Increasing the group IV concentration enhances lattice distortion and softens shear-related elastic moduli, effects that are generally associated with mechanical properties in refractory alloys. The VEC dependence of the dislocation-dipole formation energy persists after normalizing by the continuum-elastic dipole energy computed from the composition-dependent elastic constants, indicating a contribution beyond the linear-elastic response. A qualitatively similar trend is obtained in the chemically averaged VCA model, showing that such a trend can arise in the absence of explicit local chemical fluctuations. Direct electronic-structure evidence is still required to establish its microscopic origin. A Nye-tensor analysis of all dislocation configurations shows that the core broadens continuously with group IV content, with no composition threshold, while its sensitivity to the local chemical environment grows by an order of magnitude. Together, these results quantify how the defect energetics of a BCC medium-entropy alloy vary with group IV content and valence electron concentration, while showing that these quantities describe defect formation rather than dislocation mobility, strength, or ductility.

## IMPACT STATEMENT

This study quantifies how group IV content governs the elastic anisotropy and dislocation energetics of the refractory medium-entropy alloy NbTiZr, revealing a screw-dislocation core that broadens continuously with composition.

## GRAPHICAL ABSTRACT

**Figure:**
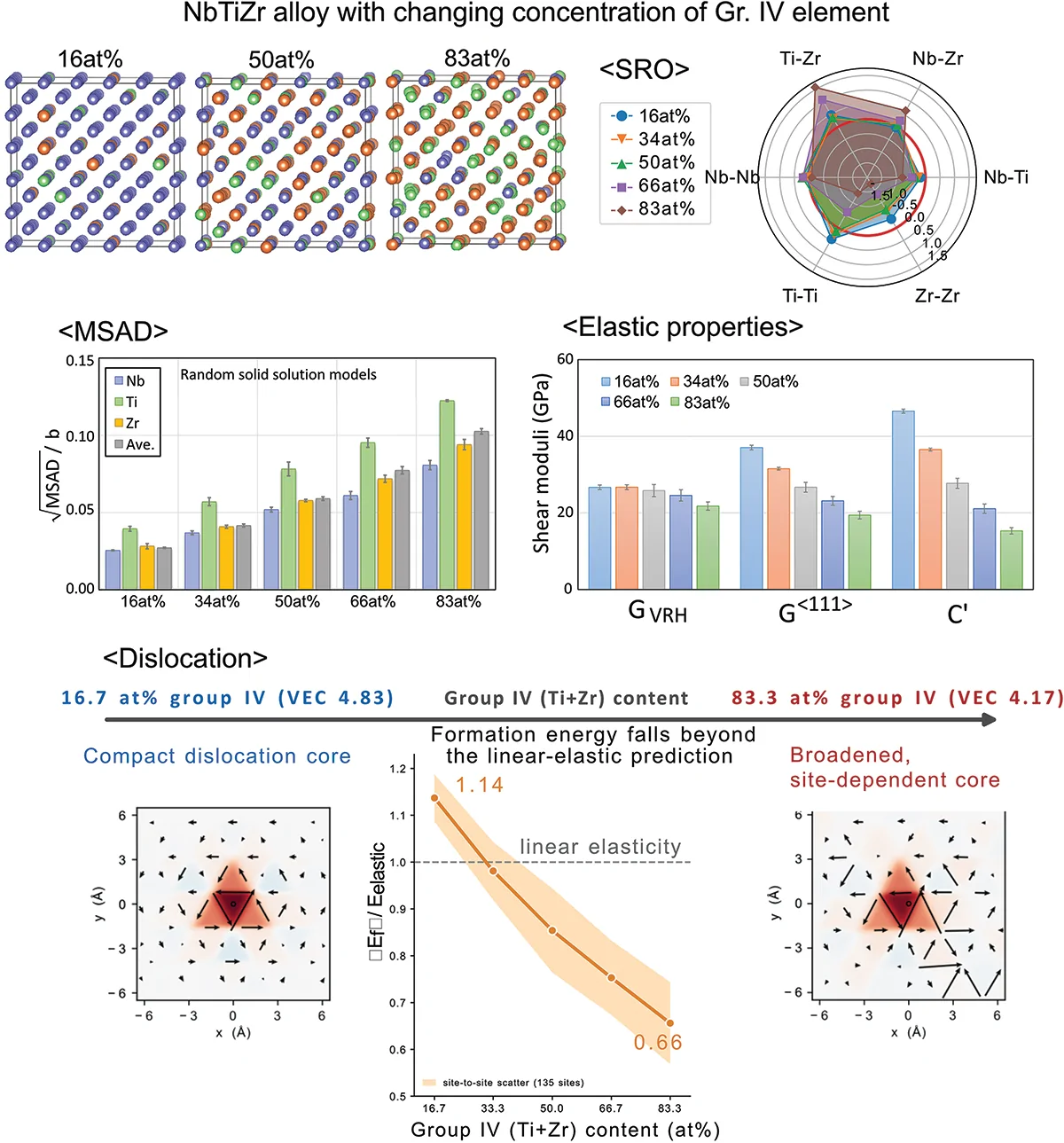

## 1. Introduction

Unlike conventional alloys based on a single principal element, high-entropy alloys (HEAs) containing multiple elements in near-equiatomic ratios have high configurational entropy, which can in certain cases result in single-phase solid-solution alloys. In particular, body-centered cubic (BCC) structures are found in refractory HEAs (RHEAs) composed of early transition metals. Among these, equiatomic TiZrHfNbTa, VNbMoTaW and their subsystems have been actively investigated as representative systems that have the potential for high strength [1–6].

While some RHEAs exhibit high strength at elevated temperatures, they often suffer from limited ductility at room temperature due to ductile-brittle transition. For example, the high-melting-point VNbMoTaW alloy (approximately 500°C higher than TiZrHfNbTa) exhibits poor ductility at room temperature even in compression [7]. In contrast, TiZrHfNbTa shows ductility in both compression and tension [8–10]. This ductility has been attributed to the nature of screw dislocation motion inherent to the BCC crystal structure. Understanding the atomic-scale core structures and mobility of dislocations is therefore essential for a fundamental understanding of the plastic deformation mechanisms in RHEAs and for establishing a foundation for optimizing their mechanical properties.

Recent experimental and computational studies, including transmission electron microscopy (TEM) observations and first-principles calculations, have provided important insights into the deformation behavior of RHEAs [11,12]. TEM studies have reported complex dislocation structures, while atomistic simulations and first-principles calculations have explored the effects of chemical disorder and electronic interactions on dislocation core structures. However, the influence of the local chemical environment and electronic structure on dislocation behavior remains insufficiently understood. This knowledge gap continues to be a major barrier to the predictive design of the mechanical properties of multi-principal-element alloys.

Most RHEAs are composed of elements from groups IV, V, and VI, and a previous study on RHEAs and refractory medium-entropy alloys (RMEAs) suggested that the addition of group IV elements (correlated with valence electron concentration: VEC) contributes to enhanced ductility [10], whereas a more recent study exhibited the opposite trend [13]. Our comparative study using experiments and computational approaches on equiatomic TiZrHfNbTa and VNbMoTaW demonstrated that the addition of group IV elements significantly affects the elastic moduli and the unusual slip behavior of dislocations [12]. Previous research has also shown that increasing the number of constituent elements in RHEAs does not necessarily improve the thermal stability of single-phase solid-solutions or their mechanical properties; rather, the careful design of both the type and concentration of constituent elements is critical. The apparent conflict between refs. [10] and [13] regarding the direction of the group IV/ductility trend implies that ductility is controlled by multiple competing processes-dislocation nucleation and glide, twinning, and possibly deformation-induced phase transformation-rather than by any single defect-energetics descriptor.

In the present study, to elucidate the effect of elemental composition on fundamental mechanical properties in RHEAs, we focused on the ternary medium-entropy alloy (MEA) NbTiZr and examined the impact of varying the concentration of group IV elements on its properties. NbTiZr represents one of the most frequent combinations of the three elements commonly used in RHEAs [2] and serves as a prototype for quaternary alloys such as HfNbTiZr [14–18], and quinary alloys such as TiNbTaZrMo [19] and NbTiZr(MoTa)0.1 [20], which are BCC-structured HEAs with potential biomaterial applications. Furthermore, equiatomic NbTiZr appears as a solid solution in the ternary Nb–Ti–Zr system [21], and has been the subject of prior investigations [22,23]. Owing to its high strength derived from significant lattice strain [22], favorable strength–ductility balance [2], and irradiation resistance [23], NbTiZr is also considered a promising candidate for nuclear structural materials. Beyond its technological relevance, the Nb–Ti–Zr alloy family provides an ideal model system for investigating the respective roles of electronic structure, lattice distortion, and elastic response in refractory alloys. Because the group IV content can be systematically varied while preserving a common BCC refractory matrix, this alloy family enables controlled tuning of the valence electron concentration (VEC) and lattice distortion within a relatively simple chemical space. This relatively simple alloy family enables the coupled evolution of composition, elastic response, and lattice distortion to be examined systematically, although the present composition path does not isolate these variables independently. Here, we performed a comprehensive first-principles analysis of NbTiZr, considering macroscopic fundamental properties such as elastic moduli, as well as HEA- and MEA-specific features including short-range order (SRO) formation, lattice distortion, unstable stacking fault energies (SFE), and dislocation formation. Special quasi-random structure (SQS) calculations are used for a set of fundamental properties including the defect energetics of the ternary alloy, while virtual crystal approximation (VCA) calculations are also used to examine the formation energy of dislocations of the corresponding chemically averaged system; on this basis we assess how far the composition dependence of the formation energy of dislocations can be accounted for by the elastic response and local lattice distortion. This allows us to characterize systematically how the constituent elements influence the defect energetics of the alloy.

## 2. Methods

### 2.1. Atomic models

Refractory Nb-Ti-Zr MEAs were taken as typical examples of RMEAs containing group V (Nb) and group IV elements (Ti and Zr). First-principles calculations were carried out to investigate fundamental mechanical properties, formation of short-range ordering, and dislocation core features. Atomic models of NbTiZr alloys with different concentrations of the constituent elements (Nb–8.3Ti–8.3Zr, Nb–16.7Ti–16.7Zr, Nb–16.7Ti–33.3Zr, Nb–33.3Ti–33.3Zr and Nb–33.3Ti–50.0Zr, in at.%) were considered. These correspond to group IV contents *c*_IV_ of 16.7, 33.3, 50.0, 66.7 and 83.3 at.% and, with the conventional counts Nb = 5 and Ti = Zr = 4, to VEC values of 4.833, 4.667, 4.500, 4.333 and 4.167, equally spaced at intervals of 1/6; the alloys are referred to by *c*_IV_ throughout. The integer occupations actually realized in the 250-atom and 405-atom cells, together with the VEC recalculated from them, are given in Supplementary Table 1; the deviations from the ideal VEC are at most 0.003, and the ideal values are quoted throughout the main text. We note that this composition path varies the Nb:(Ti+Zr) ratio while the group IV content and total VEC change together by construction, so the present series does not vary VEC independently of total group IV content, group IV type (Ti versus Zr), or lattice distortion. To construct a statistically random solid-solution, special quasi-random structures (SQS) were generated using the mcsqs function in the alloy theoretic automated toolkit (ATAT) [24]. At first, a 250-atom supercell with dimensions of 5 × 5 × 5 lattice units along [100], [010], and [001] was prepared to evaluate fundamental properties such as the lattice constant, elastic constants, and local lattice distortion, where five independent SQS models were used.

Subsequently, the core structures of a screw dislocation were explored within the first-principles framework. A dislocation dipole was inserted into a 405-atom supercell, with dimensions $a=5e\prime_{1}$, $b=2.5e\prime_{1}+4.5e\prime_{2}$, and $c=3e\prime_{3}$, where a coordinate system corresponding to $x=[11\overset{ˉ}{2}]$, $y=[1\overset{ˉ}{1}0]$ and z=[111] directions is defined, and the cell vectors are $e\prime_{1}=a_{0}\left[\sqrt{6},0,0\right]$, $e\prime_{2}=a_{0}\left[0,\sqrt{2},0\right]$, and $e\prime_{3}=a_{0}\left[0,0,\sqrt{3}/2\right]$, with lattice constant *a*_0_. It has been shown that fundamental properties of screw dislocations in BCC metals can be reproduced well when a dipole is arranged in the above configuration [25]. As the cumulative displacement component along the **c** direction appears according to the elasticity theory of the dislocation dipole configuration in a periodic cell [26], the component of $0.5e\prime_{3}$ is added to the supercell vector **b**, so that when the dislocation dipole is inserted, it becomes $b=2.5e\prime_{1}+4.5e\prime_{2}+0.5e\prime_{3}$. The positions of atoms in the supercell were determined according to the BCC crystal coordinate system. The actual composition ratios and VEC of the atomic models used are shown in Supplementary Table 1. Note that the SRO-containing dislocation cells were not derived from the 250-atom Monte Carlo trajectories described in Section 3.1: a separate SQS model was generated at the 405-atom dislocation cell geometry for each composition, sampled under the same Monte Carlo protocol, and the lowest-energy 800 K configuration was adopted as the SRO state. The dipole was then introduced into that fixed configuration at each of the 135 insertion sites and relaxed, exactly as for the random models, so that the two series differ only in the underlying chemical configuration. The continuum-elastic dipole energy used to scale the DFT dipole formation energy was evaluated using anisotropic linear elasticity.

### 2.2. First-principles calculations

First-principles calculations were implemented using the Vienna ab initio simulation package (VASP) [27,28]. Projector augmented wave potentials [29] were employed with the Perdew–Burke–Ernzerhof generalized gradient approximation exchange-correlation density functional [30]. The Brillouin-zone Γ-centered *k*-point samplings were chosen using the Monkhorst–Pack algorithm [31], where Γ-centered 3 × 3 × 3 and 1 × 1 × 6 grids were used for 250-atom and 405-atom models, respectively. A cutoff in plane-wave energy of 400 eV was applied using a first-order Methfessel–Paxton scheme that employed a smearing parameter of 0.1 eV. The total energy was converged for all calculations within 10^−6^ eV/atom. The relaxed configurations were obtained using the conjugate gradient method that terminated the search when the force on all atoms was reduced to 10^−2^ eV/Å. The Virtual Crystal Approximation (VCA) in QUANTUM ESPRESSO package [32] was also employed to evaluate the elastic constants and formation energy of dislocations of the virtually mixed state of the Nb-Zr alloy system. Each virtual Nb–Zr composition was selected to match the group IV content and nominal VEC of the corresponding ternary Nb–Ti–Zr SQS composition. The dislocation cells use the same geometry, dipole separation, line length, relaxation constraints, and formation-energy reference. The comparison is restricted to the trends of the two series; no VCA quantity is combined with SQS data in a single derived quantity. The optimized parameters for kinetic energy cutoff of 52 Rydberg and Γ-centered 1 × 1 × 18 grids were used. Davidson’s iterative diagonalization and Broyden–Fletcher–Goldfarb–Shanno algorithm were applied for the self-consistent field of electronic structure and structural relaxation, respectively. Because the SQS and VCA results originate from two different codes and pseudopotential schemes (VASP/PAW versus QUANTUM ESPRESSO with mixed pseudopotentials), quantities from the two are not combined in any single derived quantity; a direct cross-code benchmark of a fundamental property was therefore carried out before comparing the two datasets. The core energies of the ½⟨111⟩ screw dislocation in pure Nb agree between the two codes to within 2% for the easy-, hard- and split-core configurations, as shown in Supplementary Table 2. Finally, all calculations were performed without spin polarization. This was verified rather than assumed: spin-polarized calculations on the perfect-crystal models of the equiatomic composition and of the most group IV–rich composition converge to non-magnetic ground states with zero local and total magnetic moments, and are degenerate with the non-spin-polarized results to within 0.04 meV/atom. This is consistent with Nb, Ti and Zr being non-magnetic in their elemental BCC forms, and with BCC alloys of this family, including Nb–Ti and Nb–Zr, being established type-II superconductors. The atomic configurations were visualized using VESTA [33] for the perfect crystal.

### 2.3. Analysis of the dislocation core structure

The core structure was characterized for every relaxed configuration by differential displacement (DD) maps [34] and by the Nye dislocation density tensor [35,36]. Both were referenced to the corresponding perfect SQS cell with identical chemical configuration and relaxation constraints; the homogeneous cell-shape difference associated with the dipole-induced tilt was removed through the mapping with *F* = *A*_dis_ · *A*_per_^−1^, and the relative displacement along the line direction is wrapped into (−|*b*|/2, |*b*|/2] to remove the spurious displacement of order |*b*| carried by bonds crossing the branch line of the screw field. Omitting the latter reduces the recovered Burgers content by a factor of about six.

The differential displacement of a bond is d*u* = [*q*_z_ − (*F* · *p*)_z_], where *p* and *q* denote the same bond in the perfect and dislocated cells. Arrows are drawn at the midpoint of each first-neighbor pair of atomic columns, parallel to the pair, scaled so that an arrow spanning the full inter-column distance corresponds to d*u* = |*b*|/3, with the Burgers vector pointing out of the projection plane.

For the Nye tensor, the lattice correspondence tensor *G*_a_ was obtained for each atom by least squares from *G*_a_ *q*_aj_ ≈ *p*_aj_ over all bonds within 1.35 times the first-neighbor distance. The 405 atoms form 135 atomic columns in ⟨111⟩ projection; *G* was averaged within each column, interpolated onto a regular 160 × 160 grid in fractional coordinates, and α_ij_ = −ε_jkl_ ∂*G*_il_/∂*x*_k_ evaluated by central differences on that grid. A regular grid is necessary because a least-squares gradient at the atomic positions is not a conservative discrete curl and underestimates ∫α_33_ d*A* by a factor of five to six. The screw component was integrated over the Voronoi domain of each core to give the cumulative Burgers content *B*(*r*) = ∫α_33_ d*A*, and the core width was defined by the radii *r*_50_ and *r*_90_ at which *B*(*r*) reaches 50% and 90% of |*b*|. The signed integral is used because the background scatter from chemical relaxation has random sign and cancels, whereas |α_33_| accumulates it with *r* and overestimates the width. The procedure recovers |*b*| to within 0.03% in all configurations.

## 3. Results

### 3.1. Formation of short-range order

Short-range order was generated by Metropolis Monte Carlo (MC) sampling on the fixed BCC lattice. Each trial move exchanged the species of a randomly chosen pair of unlike atoms and was accepted according to the Metropolis algorithm [37] with probability min[1, exp(−Δ*E*/*k*_B_*T*)], with Δ*E* evaluated by first-principles calculation at fixed atomic positions using Γ-point sampling and a 10^−4^ eV convergence threshold. Relaxation was not performed during sampling, so the sampled energetics exclude relaxation-mediated contributions to the ordering energy. One trajectory was run per composition at *T* = 300 and 800 K, comprising 16–20 and 11–15 attempted exchanges per atom respectively, with realized acceptance ratios of 14–38% and 54–72%, decreasing systematically with increasing group IV content. These lengths are one to two orders of magnitude below the 10^2^–10^3^ exchanges per atom generally required to equilibrate short-range order in a concentrated ternary, and the low acceptance at 300 K means configuration space was explored least efficiently where the ordering tendency is strongest. We therefore do not claim thermal equilibrium. Because the trajectories were finite and not demonstrably equilibrated, the configurations adopted as the SRO models were the lowest-energy configurations encountered in the respective trajectories. These configurations are extreme-value selections rather than equilibrium samples. The random-versus-SRO comparison therefore probes the sensitivity to these particular ordered configurations and does not represent a thermal average or an upper bound on the ordering effect.

The SRO formation at two temperatures (300 and 800 K) was investigated and analyzed based on the Warren-Cowley (WC) SRO parameter [38]. The local ordering parameter in terms of each pair of elements can be defined by the modification of this parameter [39]. In the present study, the following definition was used for the SRO parameter: $\Delta \delta_{ij}^{k}=N_{ij}^{k}-N_{ij,\mathrm{rand}}^{k}$, where *k* denotes *k-*th nearest-neighbor shells, $N_{ij}^{k}$ is the number of pairs between atoms of type *i* and *j* in the *k-*th shell, $N_{ij,\mathrm{rand}}^{k}$ is the expected number of such pairs in an ideally random solid solution, which is proportional to the corresponding concentrations. We emphasize that, as defined above, this quantity is the difference between the actual and the random pair counts, and is not normalized by the random expectation. It is related to the conventional Warren–Cowley parameter by Δ*n*_*ij*_ = −α_ij_ *n*_*ij*,rand_, so the two are exactly interconvertible. Note that the SRO parameter Δδ used in the present analysis has the opposite sign to the definition in ref [39]. i.e. the SRO parameter is positive when the probability of the pair is higher than the ideally random configuration as used in ref [12,40].

The SRO parameter of the first nearest neighbor pair is discussed here. The SRO parameters defined above for each composition are summarized in Figure 1, where each radar chart includes the SRO parameter of all compositions for random solid-solution and SRO-containing models after the MC simulations at 800 and 300 K. If there is no SRO formation corresponding to the ideally random solid-solution, the value should be zero, as shown by the red circle. The initial configuration prepared by SQS as shown in Figure 1(a) is confirmed to be close to this red circle. Focusing on the strong trend of SRO formation after MC simulations, it is noted that Ti-Zr and Nb-Zr pairs increase while Nb-Ti and Zr–Zr pairs decrease, and this tendency becomes stronger as the temperature decreases. The number of Ti–Ti and Nb–Nb pairs increases or decreases depending on the concentration of group IV elements. It was confirmed that pairs containing group IV elements are strongly involved in SRO formation, and that this contributes to a decrease in the energy of the system as shown in Supplementary Figure 1.

**Figure 1.**
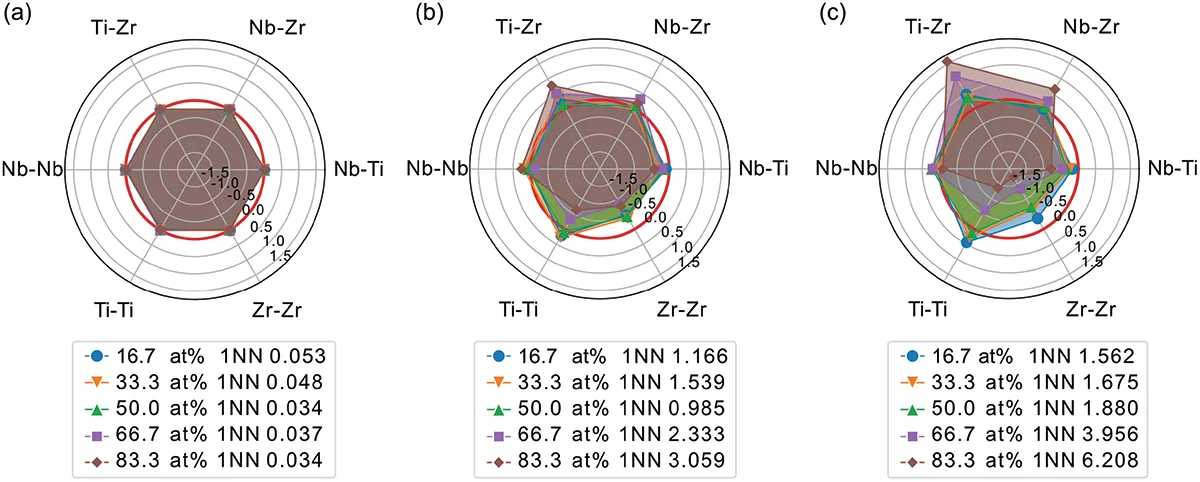
SRO parameters for atomic pairs within the first nearest-neighbor shell. SRO parameters are shown for the initial random solid-solution model (a), MC simulations at *T* = 800 K (b), and at *T* = 300 K (c). Zero values, indicated by red circles, correspond to the ideal random solid-solution state, whereas positive and negative values (outside and inside the circles) indicate enhanced and reduced pair formation, respectively.

### 3.2. Lattice distortion

Lattice distortion is one of the important characteristics that influences the mechanical properties of HEAs. The δ parameter, defined by the following relation and associated with the difference in the atomic radius of the constituent elements, has been used to estimate the magnitude of lattice distortion [41].

(1) $$\delta =\sqrt{\sum_{i=1}^{n}c_{i}{\left(1-r_{i}/\overset{ˉ}{r}\right)}^{2}}\times 100,$$

where *i* denotes the type of element, *c_i_* and *r_i_* are the concentration and atomic radius of element *i*, respectively, and *r = Σ c_i_r_i_* is the concentration-weighted average atomic radius. A number of experiments have shown that the yield stress was associated well with the lattice distortion quantified by the *δ* parameter [42–44]. However, this approach has a few problems including the fact that some of the constituent elements have crystal structures that are different from the crystal structures of the alloys, so it is unclear how physically relevant the pure-element radii are to their effective radii in the alloy, and there are several different ways to estimate pure-element radii (even if there is no crystal structure change) and it is unclear which of the methods is expected to yield physically realistic predictions. Atomistic simulations have the advantage that the local lattice distortion based on the displacement of each atom in the alloy can be directly estimated without having to rely on any pure-element values. In the present study, the mean square atomic displacement (MSAD) [45] defined by the following equation was used to evaluate the local lattice distortion of the average of each element and the whole system:

(2) $$\mathrm{MSAD}=\frac{1}{N}\sum_{i=1}^{N}{\left|r_{i}-r_{i}^{0}\right|}^{2},$$

where *N* is the number of atoms, $r_{i}$ is the coordinate of atom *i* after relaxation, and $r_{i}^{0}$ is the initial lattice point. To evaluate the MSAD, full atomic relaxation was performed for both random and SRO-containing models after MC simulations at 800 K. The square root MSAD normalized by the magnitude of Burgers vector *b* ($a_{0}/2\left\langle 111\right\rangle$) is shown in Figure 2, where the MSAD values in random solid-solution and SRO-containing models are shown in Figure 2(b,c), respectively. It is commonly seen in all compositions that there are apparent differences in the MSAD depending on element type; Ti especially has larger lattice distortion than other elements as observed in our previous study for TiZrHfNbTa [12]. The average values of MSAD in terms of the concentration of group IV elements and corresponding VEC are summarized in Table 1, where the δ parameters calculated using metallic radii are also provided for comparison. Lattice distortion, as evaluated by both δ parameter and MSAD, tends to increase as the VEC decreases, that is, the addition of group IV elements has a direct influence on the increase in lattice distortion. The two measures nevertheless scale very differently across the composition range, with δ increasing by roughly 75% between 16.7 and 83.3 at.% while the MSAD increases by roughly 275%. This difference is expected, because δ is constructed from tabulated pure-element radii whereas the MSAD is obtained directly from the relaxed atomic positions and therefore also captures the local relaxation response of the actual chemical environment. We regard the MSAD as the more physically appropriate descriptor of solid-solution friction, since solid-solution-strengthening models relate the friction stress to the actual displacement field around solutes rather than to pure-element radii; we note, however, that the MSAD is comparatively insensitive to SRO formation in the present system, so it should not be assumed to capture every chemistry-related contribution to the friction stress, and direct evaluation of solute–dislocation interaction energies would be needed to settle the choice of metric quantitatively. More interestingly, the absolute value of lattice distortion is relatively large compared to FCC HEA. The average square root of MSAD reaches 5% of the Burgers vector when *c*_IV_ is at or above 50 at.% group IV in the present BCC refractory MEAs while it is only around 2% in Cantor alloy and its subsystem [46]. Another important finding is that even though the present BCC refractory MEAs exhibit a strong tendency to form SRO, the difference in MSAD between random and SRO models was not significant as shown in Figure 2(b,c). This feature is also different from that of FCC HEAs, in which the MSAD significantly decreases after SRO formation in Cantor and its subsystem [46]. In other words, the formation of SRO in these BCC refractory MEAs appears to be predominantly governed by preferences of chemical interaction while elastic strain energy and spin ordering contribute to the formation of SRO in Cantor alloy. We note, however, that because atomic relaxation was excluded during the Monte Carlo sampling used to generate the SRO-containing configurations (Section 3.1), the relative contribution of elastic strain energy to SRO formation in the present BCC MEAs could not be independently evaluated, so this interpretation should be regarded as tentative until the omitted relaxation contribution is assessed directly.

**Figure 2.**
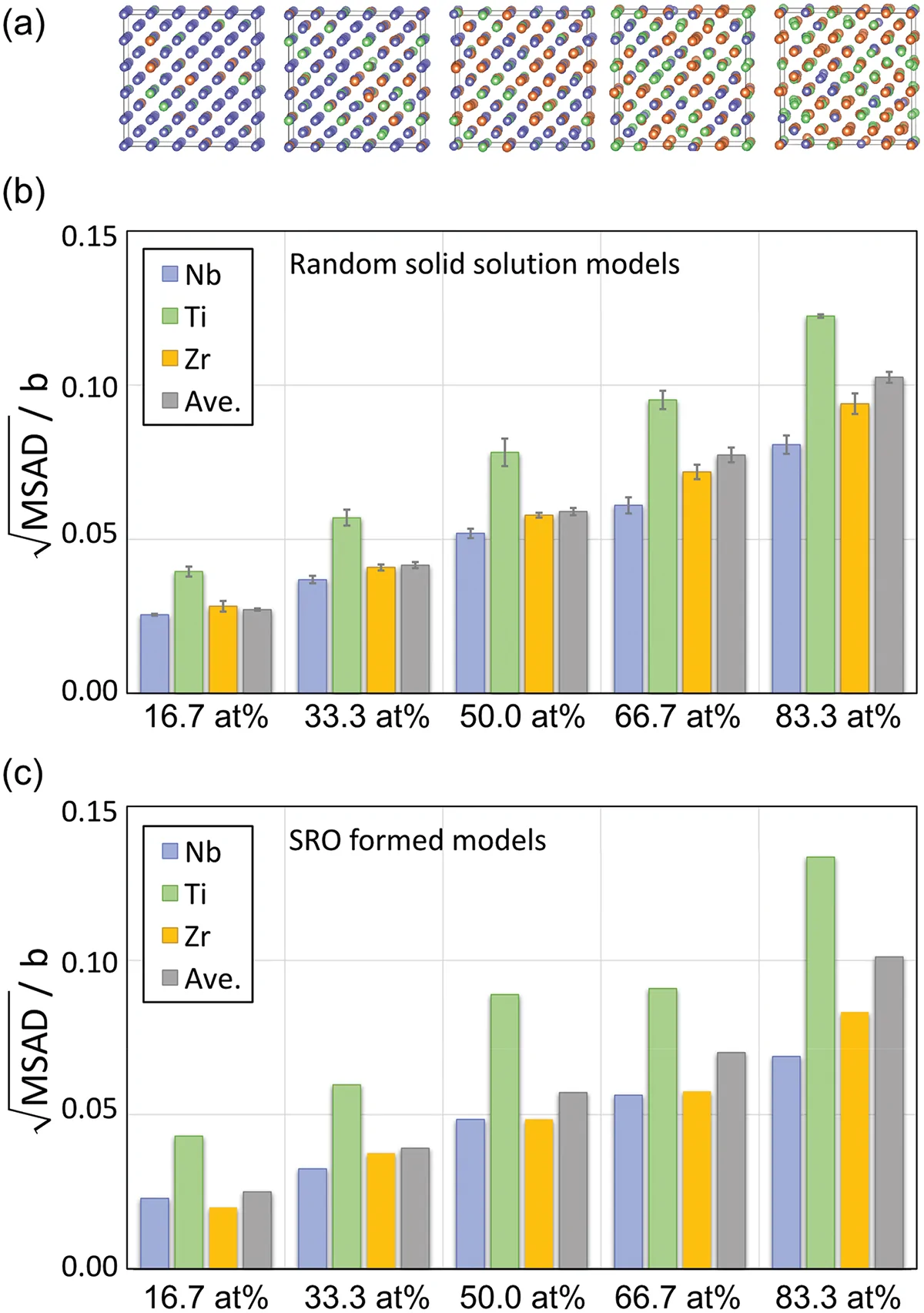
Local lattice distortion quantified by the mean squared atomic displacement (MSAD). The square root of the MSAD, normalized by the Burgers vector magnitude, is shown for each constituent element and for the system average. Representative atomic configurations after full structural relaxation of random models at each concentration (a). The dependence of lattice distortion on group IV element concentration for random solid-solution models (b) and for SRO models at 800 K (c). Data for the random models are averaged over five independent samples, with error bars representing the standard deviations.

**Table 1.** Local lattice distortion quantified by the δ parameter and the average MSAD.

*c*_IV_	VEC	δ	$\sqrt{\mathrm{MSAD}}/b\times 100$
16.7 at.%	4.83	2.70	2.71
33.3 at.%	4.67	3.31	4.16
50.0 at.%	4.50	3.97	5.25
66.7 at.%	4.33	4.17	7.73
83.3 at.%	4.17	4.75	10.26

### 3.3. Fundamental mechanical properties

We examined how group IV elements influence the bulk properties of the alloys. Table 2 summarizes the lattice and elastic constants calculated at 0 K for the atomic models obtained after structural relaxation of the random solid-solution models used above. The corresponding VCA results are provided in Supplementary Table 3. At matched nominal VEC values, *a*_0_, *C*_12_, and *B* agree within 6% between the SQS and VCA series, while *C*_11_ agrees within 14%; by contrast, *C*_44_ differs by up to 55%. The VCA shear response is therefore not treated as a quantitative representation of the ternary alloy. The lattice constant (*a*_0_) shows a slight increasing trend with decreasing VEC, but it was confirmed to agree well with the weighted average of the lattice constants of the constituent BCC elements (3.248, 3.574, and 3.309 Å for Ti, Zr, and Nb, respectively) based on their concentrations. In other words, the lattice constant of the BCC‑structured high-entropy alloys follows Vegard’s law.

**Table 2.** Lattice and elastic constants of the BCC refractory MEAs with different concentrations of group IV elements.

*c*_IV_	VEC	*a*_0_ (Å)	*C*_11_ (GPa)	*C*_12_ (GPa)	*C*_44_ (GPa)	*B* (GPa)
16.7 at.%	4.83	3.334	218.1	125.0	18.0	156.0
33.3 at.%	4.67	3.350	189.5	116.5	21.6	140.9
50.0 at.%	4.50	3.396	160.4	105.0	24.6	123.4
66.7 at.%	4.33	3.387	142.0	99.8	27.2	113.9
83.3 at.%	4.17	3.438	123.1	92.5	27.6	102.7

The elastic constants vary markedly depending on the concentrations of the constituent elements, where the change in each component depending on composition was visualized by bar chart in Supplementary Figure 2. While the *C*_11_ and *C*_12_ components decrease with increasing concentration of group IV elements, the *C*_44_ component increases. Figure 3 shows the changes in various shear moduli as a function of the concentration of group IV elements. Here, *G*_VRH_ is the Voigt–Reuss–Hill average shear modulus, expressed as the arithmetic mean of the Voigt and Reuss averages and defined together with the tetragonal shear modulus *C*′, which is defined as *C*′ = (*C*_11_ − *C*_12_)/2 and corresponds to the resistance to {110} shear along the 〈110〉-type direction lying in that plane. Details are provided in the Supplementary Discussion. *G*^<111>^ represents the shear modulus on the slip plane of the BCC structure and corresponds to the component of the elastic-constant tensor obtained by an orthogonal transformation of the elastic-constant tensor defined in the $\left[1\overset{ˉ}{1}0]-[11\overset{ˉ}{2}]-[111\right]$ coordinate system. *G*_VRH_ shows only a weak dependence on group IV concentration, whereas both *G*^<111>^ and *C*′ decrease markedly with increasing content. Previous studies have shown that elastic constants and mechanical properties correlate well with VEC [47–51], and the present results are consistent with those results. In particular, since the *C*_11_–*C*_12_ component constitutes one of Born’s stability criteria, these results indicate that the addition of group IV elements drives the alloy closer to elastic instability. It is worth noting that this softening is strongly anisotropic: while *C*′ falls from 46.6 to 15.3 GPa across the composition range, *C*_44_ increases from 18.0 to 27.6 GPa, so the Zener anisotropy ratio *A* = *C*_44_/*C*′ rises steeply with group IV content, from 0.39 at 16.7 at.% to 1.80 at 83.3 at.%, crossing unity within the series. Since the elastically compliant directions of a cubic crystal are determined by the sign of *A* −1, this crossing implies that the softest directions invert from ⟨111⟩-type at the Nb-rich end to ⟨100⟩-type at the group IV–rich end. Because {110} and {112} shear resistances are weighted differently by *C*′ and *C*_44_, this growing anisotropy may bias the relative ease of shear on different plane families and hence influence slip-system selection and the propensity for non-screw or non-planar slip. We note this as an interpretive possibility rather than a demonstrated result, since the present study does not compute resolved shear resistances on competing slip planes; a systematic comparison of {110} and {112} glide resistances across the composition series would be required to test it.

**Figure 3.**
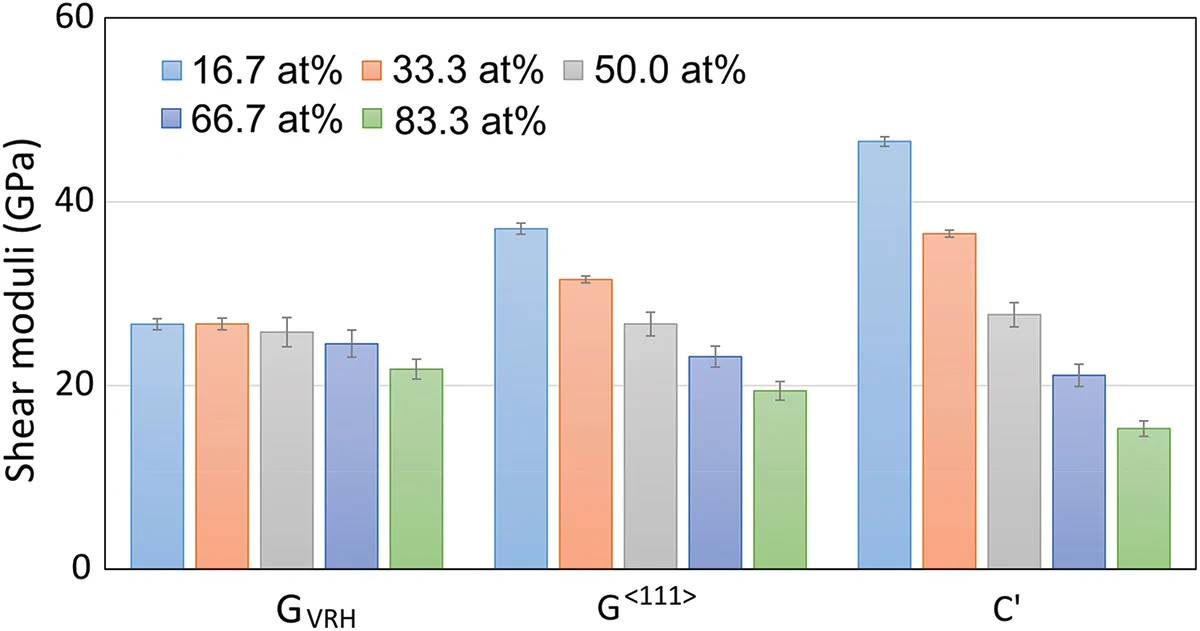
Shear modulus components of the BCC refractory MEAs as a function of concentration of group IV elements. Each shear modulus component was obtained by orthogonal transformation of the elastic constants *C*_ij_ listed in Table 2.

In addition to bulk properties and lattice distortion, the unstable SFE and the twin formation energy, which govern the deformation behavior of HEAs and MEAs, were systematically evaluated at 0 K. The atomic models used in this study consist of six layers along the ⟨110⟩ direction, allowing six distinct configurations of SF models to be constructed by inserting a stacking fault consecutively between pairs of layers. Similar to the case of lattice distributions, the unstable SFE and the formation energy of {112} twins, calculated for both the random solid-solution models and the SRO-containing models obtained from MC simulations at 800 K, are shown in Figure 4. Here, the unstable SFE (γ_us_) is defined as the maximum value along the generalized SFE path shown in Supplementary Figure 3, which also indicates that no stable stacking fault is formed at any of the compositions, that is, no stable fault exists. Both γ_us_ and the twin-formation energy decrease with increasing group IV content, reflecting their dependence on elemental type and concentration. In contrast, when focusing on the effect of SRO formation, the unstable SFE is largely insensitive to SRO formation. The twin formation energy (*E*_twin_) exhibits a similar decreasing trend with increasing group IV concentration as the unstable SFE, but is more strongly influenced. The *E*_twin_ data show considerable scatter in both the random and SRO models, with error bars spanning approximately 0.025-0.03 J/m^2^ at 50 and 66 at.%, which can be attributed to the limited number of atoms (15 atoms) on the twin plane. In other words, the local arrangement of elemental species and the presence of SRO near the twin plane have a significant influence on the formation energy. Given that this configurational scatter is comparable to the composition-to-composition variation itself, the apparent decrease in twin-formation energy with group IV content cannot at present be attributed to an intrinsic VEC effect; larger twin-plane areas and a substantially greater number of independent chemical configurations would be needed to separate an intrinsic compositional trend from local-chemistry noise, and we accordingly present this trend as tentative.

**Figure 4.**
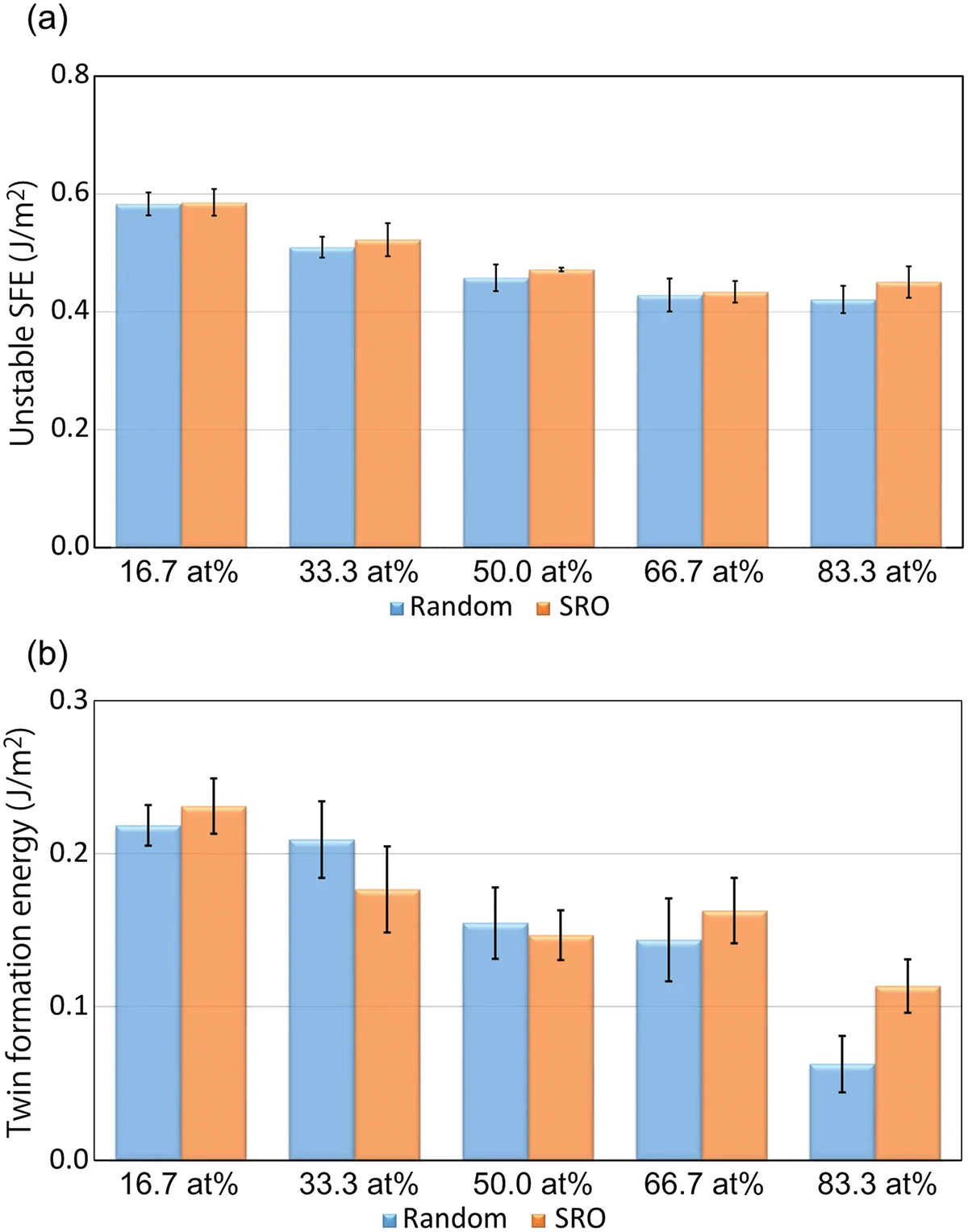
Unstable stacking fault energy and twin formation energy of BCC refractory MEAs. The unstable SFE and twin formation energy are shown for the BCC refractory MEAs with varying concentrations of group IV elements for both random solid-solution and SRO models. Error bars indicate standard deviations over the six fault positions.

### 3.4. Dislocation core features from first-principles calculations

Finally, the characteristics of dislocations for each composition were examined. Figure 5 shows the α_33_ field with the differential displacement map superimposed, for the configuration whose *r*_90_ equals the median of the 135 sites in each composition; it illustrates the core structure, while the quantification itself uses every configuration. The dislocation core structures were visualized using our visualization code based on Nye tensor and differential displacement vector. Because the core structure in a chemically disordered alloy varies with the local atomic environment, as does the formation energy (Figure 6), images of individual configurations cannot by themselves establish a composition-controlled transition. We therefore quantified the core structure for every configuration using the Nye tensor, following the procedure of Section 2.3, and the resulting cumulative Burgers content is shown in Figure 7. The radius enclosing 90% of |b| increases monotonically from 0.90|b| at 16.7 at.% group IV to 1.21|b| at 83.3 at.%, and r_50_ from 0.48|b| to 0.60|b|, so the core does broaden with group IV content, in contrast to the prediction of the linear elastic solution [26], which suggests little difference in core width among constituent elements. The broadening is, however, continuous: there is no step between adjacent compositions, and the distributions of r_90_ remain unimodal at every composition, so we find no evidence for a compact-to-spread transition at a composition threshold, nor for two coexisting core structures. The more pronounced effect is the growth of the site-to-site scatter: the standard deviation of r_90_ increases along the series, from 0.055 to 0.72 Å, and at 83.3 at.% group IV r_90_ ranges from 2.6 to 6.5 Å across the 135 sites of a single chemical configuration. Within each composition, at fixed mean chemistry, the core width correlates negatively with the local Nb fraction within 6 Å of the core (Spearman ρ = −0.17 to −0.47) and with the dipole formation energy (ρ = −0.23 to −0.40), so that more widely spread cores occur in locally group IV–rich environments and are associated with lower formation energies. Considering the dislocation picture based on the Peierls–Nabarro model [52,53], both a reduction in the elastic constants and a decrease in the unstable SFE act to promote the expansion of the dislocation core, consistent with the continuous broadening observed here.

**Figure 5.**
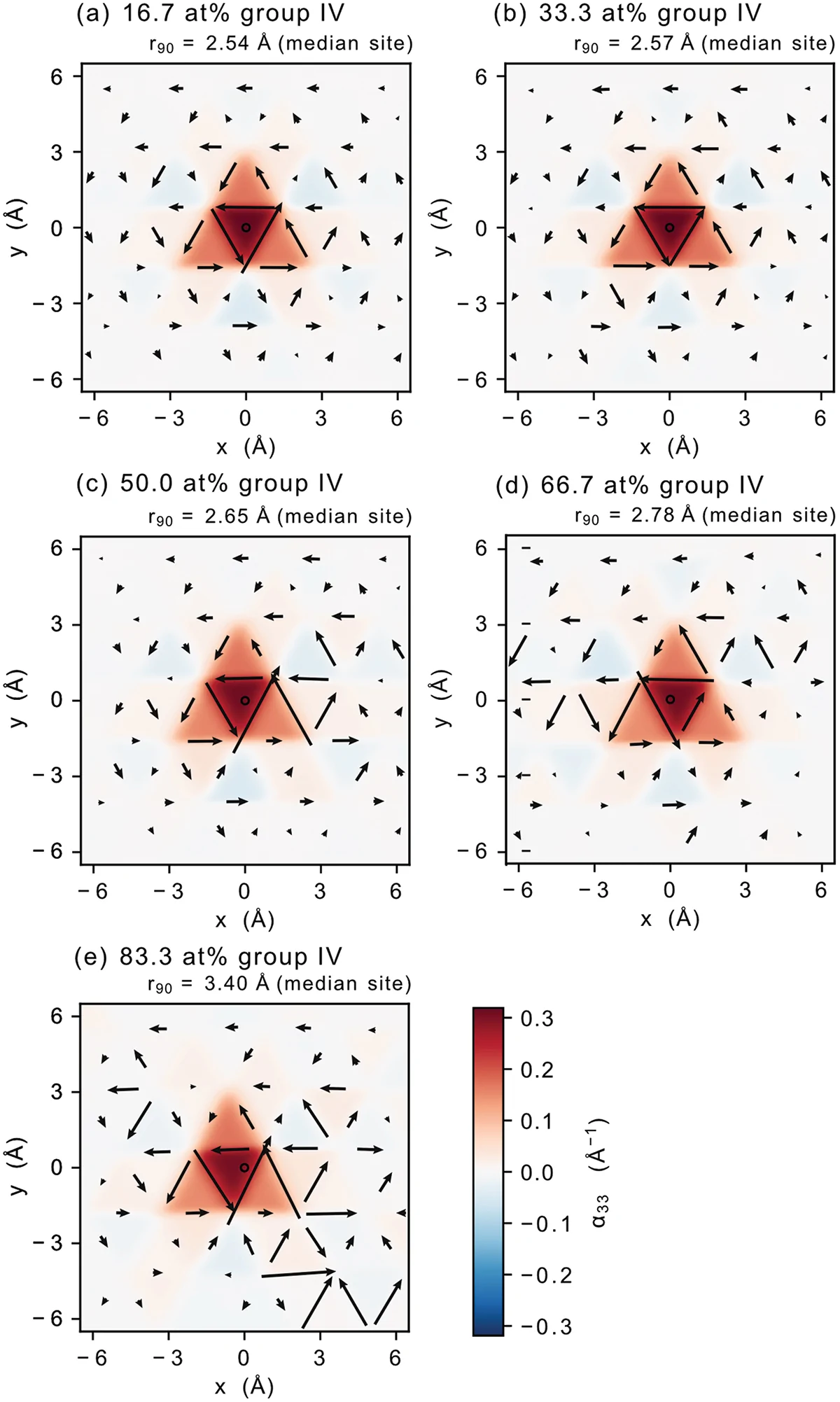
Nye density α_33_ with the differential displacement field superimposed, for the ⟨111⟩ screw core on {110} plane. Each panel is the median-*r*_90_ configuration of that alloy’s 135 insertion sites in random-state configurations; the color scale is common. Arrows give the [111] relative displacement of each first-neighbor column pair, referenced to the perfect cell of identical chemistry, with the dipole’s homogeneous shear removed and displacements wrapped into (−|b|/2, |b|/2); a full inter-column arrow corresponds to |b|/3, and b = (*a*/2)⟨111⟩ points out of the page.

**Figure 6.**
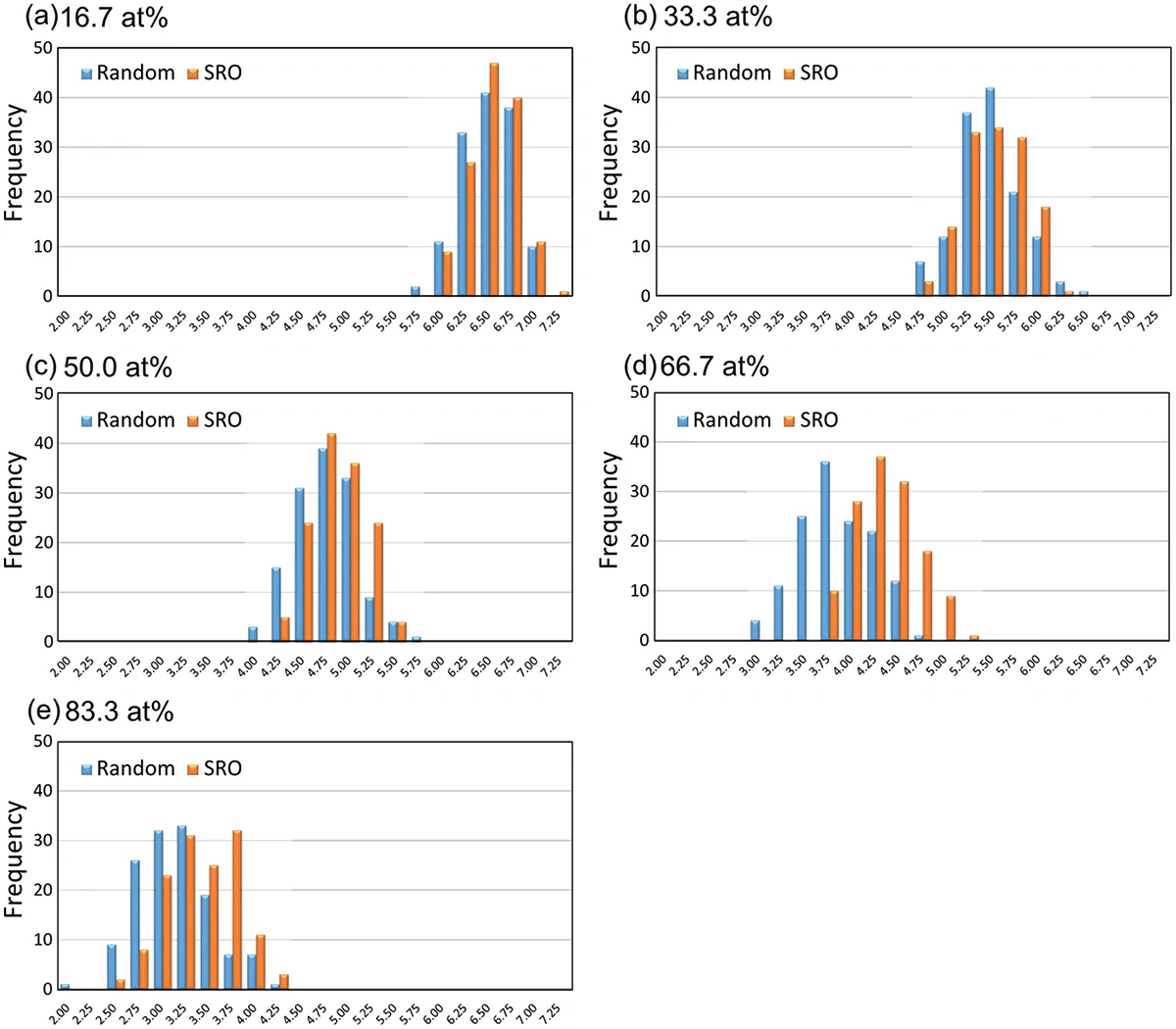
Frequency distribution of formation energy of dislocations. Frequency distributions of the energy are shown for 135 screw dislocation dipole configurations in MEAs, with and without SRO, at each concentration of group IV elements.

**Figure 7.**
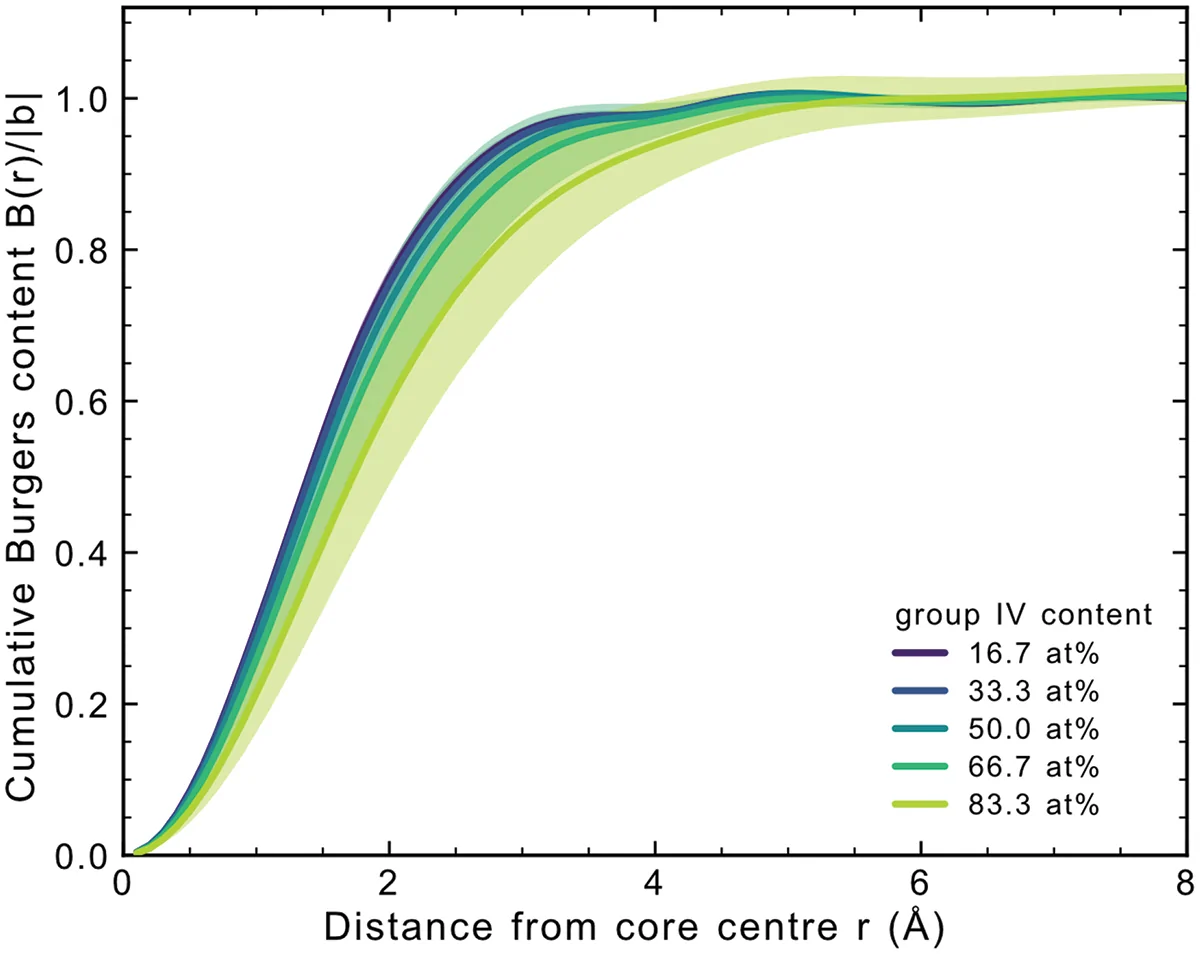
Cumulative Burgers content *B*(*r*)/|**b**| as a function of distance *r* from the core center, where *B*(*r*) is the signed integral of the Nye density α_33_ over a disc of radius *r*. Solid lines are means over the 135 insertion sites in each random-state configuration; shaded bands are ±1 standard deviation across those sites. All curves saturate at unity by *r* ≈ 5 å, confirming that the topological charge of a single dislocation is recovered.

The model used above contains 135 available sites for embedding a dislocation dipole. For each atomic model at a given concentration – both in the random solid-solution state and the SRO-formed state – we introduced dislocation dipoles at all 135 sites and evaluated the energies after structural relaxation. The formation energy at each site is defined as the total energy of the fully relaxed dislocated cell minus that of the corresponding perfect cell having the identical chemical configuration, the same lattice vectors apart from the dipole-induced tilt component described in Section 2.1, and the same relaxation constraints; energies are reported in eV per dipole for the 6b dislocation line length of the present cell, and the corresponding values per unit dislocation length are obtained by dividing by 6*b*. Two limitations of this sampling scheme should be stated explicitly. First, translating a single dipole through the 135 inequivalent insertion sites within one periodic chemical configuration probes 135 distinct local chemical environments but does not constitute 135 statistically independent alloy realizations, so the resulting distributions characterize local-environment variability rather than full configurational statistics. Second, the present energies have not been tested for convergence with respect to dipole separation and supercell size, and the elastic normalization of Table 3 becomes increasingly sensitive as the alloy approaches the Born stability limit at high group IV content; such a convergence study lies beyond the computational resources available here, and we accordingly make no claim of statistical significance for the composition dependence. The formation energies of dislocations introduced at all sites in random solid-solution and in the SRO-formed state are summarized in Supplementary Table 4. Figure 6 shows the energy distributions for formation energies within a given energy range using data shown in Supplementary Table 4, where the horizontal axis represents the dipole energy, corresponding to a dislocation line length of 6b. Focusing on the formation energy of dislocations in the random versus SRO-containing states at each concentration, the mean formation energy over the 135 sites is higher in the SRO-containing state than in the random state at 16.7, 33.3, 66.7, and 83.3 at.% group IV content, but slightly lower at 50.0 at.%, so the trend is not strictly monotonic across the full composition range and the effect at 16.7 and 33.3 at.% is small relative to the width of the site-to-site distributions; quantifying the difference against the width of the distributions gives Cohen’s d = 0.13, 0.18, −0.08, 1.41 and 0.76 at 16.7, 33.3, 50.0, 66.7 and 83.3 at.% group IV, and we therefore restrict the claim of an SRO-induced increase in formation energy to 66.7 and 83.3 at.%, where the effect is large relative to the scatter. Because the random and SRO models are different chemical configurations, a given site index does not denote the same local environment in the two sets, and the two are compared as independent samples rather than as paired data; we do not quote *p* values, since the 135 sites are not independent alloy realizations. Where present, this feature is qualitatively consistent with previous reports on the excess formation energy associated with the degree of SRO in MoNbTaW [54]. In addition, with increasing concentration of group IV element, the energy of dislocations decreases markedly.

**Table 3.** Dislocation-dipole formation energies for the random and SRO models, normalized by the anisotropic periodic elastic solution.

c_IV_ (at.%)	*|b|* (Å)	⟨*E*_f_⟩ random	⟨*E*_f_Ef⟩ SRO	*E*_elastic_	*E*_f_*/E*_elastic_
		(eV per dipole, 6*b*)	(eV per dipole, 6*b*)	(eV per dipole, 6*b*)	
16.7	2.887	6.369 ± 0.281	6.404 ± 0.255	5.603	1.137 ± 0.023
33.3	2.901	5.324 ± 0.334	5.381 ± 0.312	5.425	0.981 ± 0.020
50.0	2.941	4.515 ± 0.473	4.481 ± 0.311	5.284	0.854 ± 0.019
66.7	2.933	3.709 ± 0.390	4.221 ± 0.334	4.927	0.753 ± 0.017
83.3	2.977	3.008 ± 0.400	3.303 ± 0.372	4.585	0.656 ± 0.015

Energies are reported per dipole for the total dislocation line length of 6*b* in the supercell. ⟨*E*_f_⟩_random_ is the mean over 135 insertion sites, and ±denotes the standard deviation across those sites, not the uncertainty of the mean. The ratio *E*_f_/*E*_elastic_ is calculated using ⟨*E*_f_⟩_random_. *E*_elastic_ uses the full elastic tensor of each composition (Table 2); the uncertainty in the ratio propagates an estimated 2% uncertainty in those constants.

Since the self-energy of a dislocation scales linearly with the shear modulus, these results may at first glance appear to simply reflect the concentration dependence of the shear modulus. Moreover, the formation energies of dislocations inherently contain contributions from both elastic properties and lattice distortion associated with chemical disorder, making it challenging to establish a direct link between dislocation stability and electronic structure. Scaling the DFT dipole formation energy by the corresponding continuum-elastic energy tests whether the composition dependence follows linear-elastic scaling. We emphasize that this operation does not remove the elastic contribution, does not separate the lattice-distortion contribution, and does not provide a rigorous decomposition into core and far-field terms; any residual trend therefore identifies a contribution not captured by linear elasticity rather than an isolated electronic effect. In the following we apply this normalization and examine what remains.

The introduction of dislocations within the periodic cell is accomplished through application of a continuum linear elastic theory solution for the periodic dislocation dipole array [26]. The distortion field caused by periodic distribution of dislocations is expressed as a Fourier series and the elastic energy per unit length of the supercell can be expressed as follows:

(3) $$E_{\mathrm{elastic}}=\frac{1}{2}A_{c}C_{\mathrm{ijkl}}\sum_{G}{\tilde{\Delta }}_{\mathrm{ij}}\left(G\right)\;{\tilde{\Delta }}_{\mathrm{kl}}^{*}\left(G\right),$$

where $\Delta \left(r\right)$ is the distortion tensor at point r, G is the reciprocal lattice vectors, and $\tilde{\Delta }\left(G\right)$ is the Fourier coefficients describing the distortion tensor. *A*_*c*_ is the area of the supercell containing the dislocation dipole and $C_{\mathrm{ijkl}}$ the elastic constants of the material, each of which was summarized in Table 2. Details of the procedure can be found in the Supplementary Information of ref [55]. Using this method, the solution of elasticity theory for a dislocation dipole can be obtained for the case in which the lattice constant, elastic constants, and periodic boundary cell are identical to those used in the first-principles calculations. Table 3 summarizes the values obtained by normalizing the average of the dipole energy from the first-principles calculations with the solution of elasticity theory. Even after normalization by continuum elasticity theory, the mean *Ef*/*E*_elastic_ ratio decreases monotonically as the concentration of group IV elements increases (Table 3). The decrease across the full series, from 1.137 to 0.656, is five to ten times the site-to-site scatter of the ratio, whereas adjacent compositions differ by only 0.10–0.16, one to two times that scatter, so we do not regard individual adjacent pairs as resolved; consistent with the sampling limitation stated above, we make no claim of statistical significance for the composition dependence, since establishing formal confidence intervals would require independent alloy realizations that the present dataset does not contain. The residual trend nevertheless suggests that factors other than the elastic constants used in the normalization, which are themselves correlated with VEC, contribute to the reduction of the formation energy of dislocations; possible candidates include lattice-distortion-related strain-energy release in the perfect crystal and, potentially, electronic-structure effects, the latter of which we cannot isolate with the present dataset.

In HEAs and MEAs, the large lattice distortion results in relatively high energy even in a perfect-crystal configuration, and, as discussed above, group IV elements tend to enhance this distortion. When the perfect crystal is already in a high-energy state, the energy released upon relaxation may partly offset the energy required to introduce a dislocation, although this strain-energy-release contribution has not been separately quantified here and should be evaluated directly in future work rather than inferred from MSAD alone.

The residual, elasticity-normalized VEC trend reported in Table 3 is consistent with, but does not on its own establish, an electronic-structure contribution. To test whether it survives the removal of chemical fluctuation, we repeated the dislocation calculations for the virtual Nb–Zr system within the VCA; the results are given in Figure 8 and Table 4. A qualitatively similar trend is obtained in the chemically averaged VCA model, showing that such a trend can arise in the absence of explicit local chemical fluctuations. Direct electronic-structure evidence, such as a site-projected density of states or bonding analysis at the dislocation core, is still required to establish its microscopic origin.

**Figure 8.**
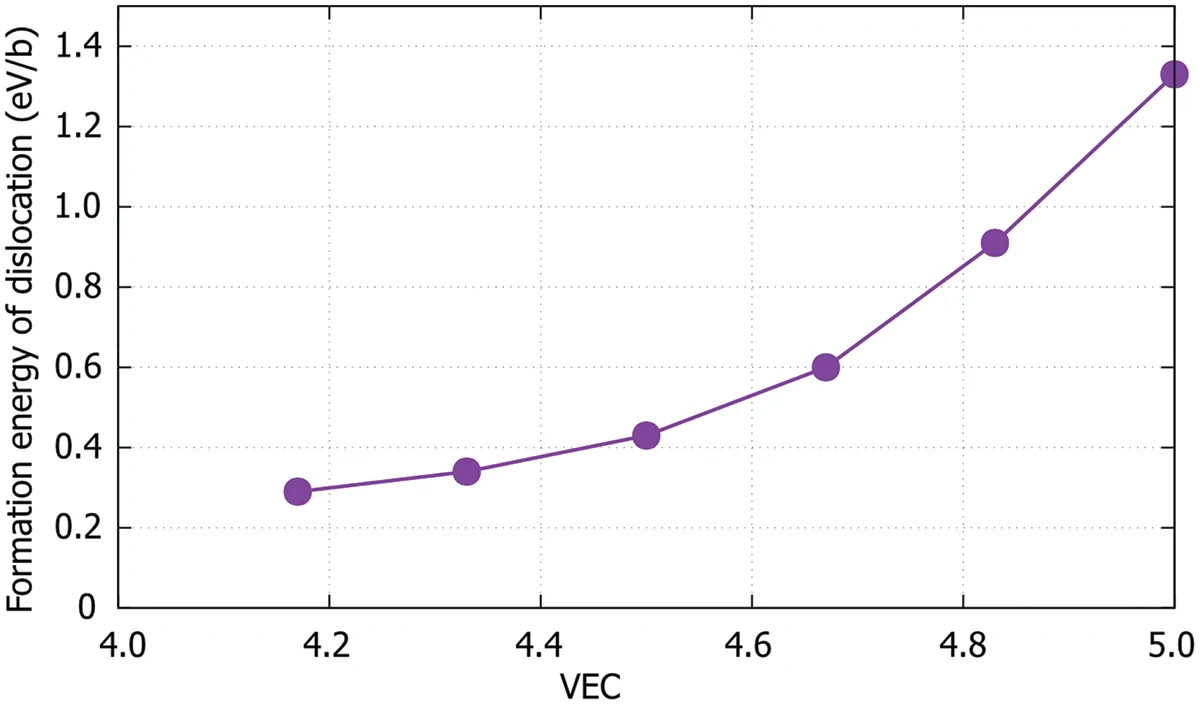
Formation energy of dislocation in terms of VEC calculated by VCA.

**Table 4.** Formation energy of dislocation calculated by VCA, normalized by the dipole energy through linear elasticity theory using elastic constants in Supplementary Table 3.

*c*_IV_	16.7 at.%	33.3 at.%	50.0 at.%	66.7 at.%	83.3 at.%
*E_f*/*E*_Elastic_	1.24	0.95	0.89	0.74	0.41

Addition of group IV element significantly reduces the unstable SFE γ_us_, which facilitates dislocation spreading and consequently reduces the strain energy caused by dislocations. Taken together, these effects indicate that in BCC solid-solution alloys containing high concentrations of group IV elements, such as TiZrHfNbTa, several factors – namely the low shear modulus, low unstable SFE, and high lattice distortion – contribute to the reduction of the formation energy of dislocations, with a VEC-correlated residual contribution also present after elasticity normalization. Furthermore, first-principles calculations have demonstrated that band filling governs the character of ideal lattice instability in BCC transition metals and alloys, favoring a shear-like mode at lower VEC and a cleavage-like mode at higher VEC [56]. It is also well established that macroscopic slip behavior – so-called slip persistence in BCC alloys – can be characterized by the electronic structure of the *d*-bands [57]. By employing the VCA, it is possible to predict how variations in the *d*-band influence the minimum energy pathway for dislocation motion [12]. These previous findings motivate VEC as a useful compositional descriptor for deformation-related properties. However, because VEC covaries with group IV content, Ti:Zr ratio, and lattice distortion along the present composition path, the present results do not establish a causal VEC-reduction mechanism for deformation.

Experimental studies have reported that the TiZrHfNbTa alloy exhibits an anomalously high dislocation density and high activity even at room temperature [11]. This behavior has been attributed to an intrinsically low formation energy of dislocations, and the present results are consistent with that interpretation, although they do not by themselves establish it. Since screw dislocations in HEAs cannot be described as perfectly straight lines, as suggested by the presence of low-energy kink configurations [58,59], evaluating the Peierls potential within a first-principles framework remains challenging. We stress that the dislocation-dipole formation energy computed here is a defect-energetics descriptor and is not equivalent to the Peierls barrier, kink-pair nucleation enthalpy, migration barrier, or any other stress-dependent measure of dislocation mobility; the present results therefore do not by themselves demonstrate that dislocation mobility increases with decreasing VEC, and dedicated glide-resistance calculations (e.g. Peierls-potential or kink-pair energetics) are needed to test this hypothesis directly. Previous studies have associated lattice distortion with shear-modulus-normalized strength in refractory alloys [12,22,45]. The present study, however, does not calculate a friction stress or a solid-solution-strengthening contribution and therefore does not assess the strength of the present alloys. A similar interpretation has been proposed for the reported difference between TiZrHfNbTa and VNbMoTaW [12], but these alloys lie outside the present composition series. Nevertheless, these findings deepen our understanding of the composition dependence of defect energetics in RHEAs and provide a foundation for future work aimed at directly linking electronic structure to strength and ductility. Regarding simulations of dislocation motion in RHEAs, methodological developments have progressed from empirical approaches [60–62] to machine-learning–based study [40,63–65], and the integration of first-principles guided element strategies with large-scale simulations is expected to enable direct evaluation of deformation behavior in the near future.

## 4. Conclusion

In this work, we systematically mapped the effect of group IV element concentration on the defect energetics of the NbTiZr refractory medium-entropy alloy system. First-principles calculations showed that increasing the group IV content enhances lattice distortion and induces pronounced softening of the shear-related elastic moduli *C*′ and *G^⟨111⟩* while *C*_44_ increases, and that it concurrently reduces the unstable SFE and, more tentatively, the twin-formation energy. Over the same range, the screw-dislocation dipole formation energy decreases substantially. A Nye-tensor analysis of all 675 random-state dislocation configurations shows that the core broadens continuously over the same range, r_90_ increasing from 0.90|*b*| to 1.21|*b*| with no threshold between adjacent compositions, while the site-to-site scatter of the core width grows by a factor of thirteen.

After normalization by the continuum-elastic dipole energy evaluated with the composition-dependent elastic constants, the mean dislocation-dipole formation energy still decreases as the valence electron concentration decreases. Because this scaling accounts only for the trend expected from the continuum-elastic dipole energy, and does not separately isolate lattice-distortion or core contributions, the residual trend remains unexplained. Candidate origins include relaxation-mediated strain-energy release in the highly distorted perfect crystal and an intrinsic electronic-structure effect. A qualitatively similar trend is also obtained in the chemically averaged VCA model, showing that such a trend can arise in the absence of explicit local chemical fluctuations; distinguishing the remaining candidates will require direct electronic-structure descriptors at the core, such as a site-projected density of states or bonding analysis, together with a convergence assessment with respect to supercell size and dipole separation, neither of which is provided here. We therefore do not claim that electronic states near the Fermi level stabilize dislocations independently of structural disorder.

Taken together, these results provide a systematic, internally consistent map of how group IV content alters the defect energetics of a BCC refractory medium-entropy alloy, spanning short-range order, lattice distortion, elastic anisotropy, unstable SFE and twin-formation energies, and screw-dislocation formation energy.

## Supplementary Material

Supplemental Material
