# Supplementary material for: Effect of group IV content on elastic and defect properties of NbTiZr refractory medium-entropy alloys
Source: Sci Technol Adv Mater. 2026 Sep 18;27(1):2736330. doi: 10.1080/14686996.2026.2736330 (PMC13686216; doi:10.1080/14686996.2026.2736330)
Supplement: Supplementary file 1 [file TSTA_A_2736330_SM8361.docx]

**Supplementary Information**

**Effect of group IV content on elastic and defect properties of NbTiZr refractory medium-entropy alloys**

Tomohito Tsuru^1*^, Ivan Lobzenko^1^, Shu Han^2^ & Haruyuki Inui^2^

^1^ Research Center for LWR Engineering, Japan Atomic Energy Agency, 2-4 Shirakata, Tokai-mura, Ibaraki 319-1195, Japan

^2^ Department of Materials Science and Engineering, Kyoto University, Sakyo-ku, Kyoto 606-8501, Japan

**Supplementary Figures**

**Supplementary Figure 1.** Energy along the Monte Carlo trajectories of the 250-atom bulk supercells at *T* = 800 K (a) and *T* = 300 K (b). The pronounced decrease at 300 K corresponds to the short-range order shown in Figure 1. Note that several 300 K trajectories retain a residual drift of up to 0.2 eV per 1000 exchanges over their final fifth and are therefore not equilibrated.

**Supplementary Figure 2.** Anisotropic elastic constants of BCC MEAs as a function of concentration of group IV elements (the error bars represent standard deviations with five independent SQS models).

**Supplementary Figure 3.** Generalized stacking-fault energy curves for the {110}⟨111⟩ path in BCC MEAs with different concentrations of group IV elements. Each panel corresponds to *c*_IV_ = (a) 16.7 at%, (b) 33.3 at%, (c) 50.0 at%, (d) 66.7 at% and (e) 83.3 at%. The fault position was shifted successively among the six interlayer planes, denoted Layers 1–6, so that each curve shows the energy obtained when the fault is placed on a different plane within the supercell.

**Supplementary Tables**

**Supplementary Table 1.** Integer occupations, atomic fractions and valence electron concentration of the 250- and 405-atom supercells. VEC is calculated from the actual occupations (Nb = 5, Ti = Zr = 4) and differs from the ideal value of the nominal composition by at most 0.003.

(a) 250-atom supercells for lattice, elastic constants and MSAD calculations

| Nominal | *N*(Nb) | *N*(Ti) | *N*(Zr) | Nb  (at%) | Ti  (at%) | Zr  (at%) | VEC | Ideal VEC |
| --- | --- | --- | --- | --- | --- | --- | --- | --- |
| Nb83Ti8Zr8 | 208 | 21 | 21 | 83.20 | 8.40 | 8.40 | 4.832 | 4.833 |
| Nb66Ti17Zr17 | 166 | 42 | 42 | 66.40 | 16.80 | 16.80 | 4.664 | 4.667 |
| Nb50Ti17Zr33 | 125 | 42 | 83 | 50.00 | 16.80 | 33.20 | 4.500 | 4.500 |
| Nb33Ti33Zr33 | 84 | 83 | 83 | 33.60 | 33.20 | 33.20 | 4.336 | 4.333 |
| Nb17Ti33Zr50 | 42 | 83 | 125 | 16.80 | 33.20 | 50.00 | 4.168 | 4.167 |

(b) 405-atom supercells for dislocation calculations

| Nominal | *N*(Nb) | *N*(Ti) | *N*(Zr) | Nb  (at%) | Ti  (at%) | Zr  (at%) | VEC | Ideal VEC |
| --- | --- | --- | --- | --- | --- | --- | --- | --- |
| Nb83Ti8Zr8 | 338 | 34 | 33 | 83.46 | 8.40 | 8.15 | 4.835 | 4.833 |
| Nb66Ti17Zr17 | 270 | 67 | 68 | 66.67 | 16.54 | 16.79 | 4.667 | 4.667 |
| Nb50Ti17Zr33 | 202 | 67 | 136 | 49.88 | 16.54 | 33.58 | 4.499 | 4.500 |
| Nb33Ti33Zr33 | 135 | 135 | 135 | 33.33 | 33.33 | 33.33 | 4.333 | 4.333 |
| Nb17Ti33Zr50 | 67 | 136 | 202 | 16.54 | 33.58 | 49.88 | 4.165 | 4.167 |

**Supplementary Table 2.** Cross-code comparison of the core energies of the easy-, hard- and split-core configurations of the ½⟨111⟩ screw dislocation in pure Nb, calculated with VASP and with Quantum ESPRESSO.

|  | VASP | Quantum ESPRESSO |
| --- | --- | --- |
| Easy core (eV/b) | 1.32 | 1.31 |
| Hard core (eV/b) | 1.40 | 1.38 |
| Split core (eV/b) | 1.41 | 1.39 |

**Supplementary Table 3.** Lattice and elastic constants of virtual Nb–Zr alloys as a function of group IV (Zr) content, calculated by the virtual crystal approximation (VCA).

| *c*_IV_ | VEC | *a*_0_ (Å) | *C*_11_ (GPa) | *C*_12_ (GPa) | *C*_44_ (GPa) | *B* (GPa) |
| --- | --- | --- | --- | --- | --- | --- |
| 16.7 at% | 4.83 | 3.341 | 216.8 | 117.7 | 17.2 | 150.8 |
| 33.3 at% | 4.67 | 3.382 | 182.1 | 113.0 | 11.4 | 136.0 |
| 50.0 at% | 4.50 | 3.425 | 154.8 | 105.3 | 11.2 | 121.8 |
| 66.7 at% | 4.33 | 3.470 | 127.4 | 99.5 | 19.3 | 108.8 |
| 83.3 at% | 4.17 | 3.518 | 106.4 | 92.1 | 31.6 | 96.8 |

**Supplementary Table 4.** Formation energies of dislocations (eV per dipole, corresponding to a dislocation line length of 6*b*) introduced at the 135 possible insertion sites in the random solid-solution and SRO states for each composition. The SRO configurations were obtained from the 800 K MC trajectories. The mean and standard deviation are taken over the 135 sites and characterize the variation of the local chemical environment, not the uncertainty of the mean; the 135 sites are not independent alloy realizations.

|  | 16.7 at% | | 33.3 at% | | 50.0 at% | | 66.7 at% | | 83.3 at% | |
| --- | --- | --- | --- | --- | --- | --- | --- | --- | --- | --- |
| site | Rand. | SRO (800K) | Rand. | SRO (800K) | Rand. | SRO (800K) | Rand. | SRO (800K) | Rand. | SRO (800K) |
| 1 | 5.86 | 6.43 | 5.44 | 5.25 | 5.04 | 4.82 | 3.55 | 4.10 | 1.98 | 3.45 |
| 2 | 5.85 | 6.44 | 6.21 | 4.86 | 5.24 | 4.95 | 4.31 | 4.24 | 2.56 | 3.28 |
| 3 | 5.63 | 6.53 | 5.85 | 5.17 | 5.22 | 4.36 | 4.20 | 4.44 | 3.02 | 3.42 |
| 4 | 5.89 | 6.23 | 5.64 | 4.87 | 5.21 | 4.26 | 4.08 | 4.31 | 2.55 | 3.40 |
| 5 | 6.27 | 6.04 | 5.45 | 4.96 | 5.25 | 4.34 | 3.27 | 3.97 | 2.37 | 3.85 |
| 6 | 6.24 | 6.23 | 5.19 | 5.36 | 5.14 | 4.33 | 3.15 | 3.90 | 2.47 | 3.97 |
| 7 | 5.96 | 6.45 | 5.64 | 5.74 | 4.45 | 4.64 | 3.06 | 4.01 | 2.87 | 3.86 |
| 8 | 6.06 | 6.26 | 5.09 | 5.60 | 4.62 | 3.79 | 2.94 | 3.79 | 3.21 | 3.46 |
| 9 | 5.92 | 6.29 | 5.76 | 5.06 | 4.58 | 4.05 | 3.28 | 3.80 | 3.06 | 3.65 |
| 10 | 6.23 | 6.52 | 5.69 | 4.95 | 4.46 | 3.94 | 3.68 | 4.49 | 3.23 | 3.48 |
| 11 | 6.12 | 6.18 | 5.58 | 5.27 | 4.78 | 3.85 | 4.10 | 4.31 | 3.86 | 3.41 |
| 12 | 6.43 | 6.19 | 5.57 | 5.43 | 4.83 | 4.08 | 4.00 | 3.94 | 3.60 | 3.34 |
| 13 | 6.67 | 6.27 | 5.35 | 5.43 | 4.90 | 4.26 | 3.37 | 4.19 | 3.02 | 3.61 |
| 14 | 6.13 | 6.68 | 5.81 | 5.39 | 4.07 | 4.67 | 3.71 | 4.02 | 2.32 | 2.86 |
| 15 | 5.82 | 6.56 | 5.41 | 5.67 | 4.28 | 5.12 | 3.72 | 4.31 | 2.29 | 3.13 |
| 16 | 5.67 | 6.88 | 5.80 | 5.55 | 4.72 | 4.40 | 3.62 | 4.85 | 2.58 | 3.13 |
| 17 | 5.80 | 6.49 | 5.74 | 5.40 | 4.72 | 4.75 | 3.99 | 4.71 | 2.91 | 3.47 |
| 18 | 6.19 | 6.32 | 5.50 | 5.26 | 4.91 | 4.65 | 3.99 | 4.12 | 2.62 | 3.53 |
| 19 | 6.33 | 5.88 | 5.29 | 5.51 | 5.25 | 4.37 | 3.26 | 3.94 | 2.77 | 3.57 |
| 20 | 6.17 | 6.14 | 5.38 | 5.60 | 4.79 | 4.71 | 3.45 | 3.93 | 2.72 | 3.44 |
| 21 | 6.13 | 6.48 | 5.18 | 5.47 | 5.01 | 4.96 | 3.33 | 4.06 | 3.03 | 2.97 |
| 22 | 6.26 | 6.41 | 5.08 | 4.93 | 5.50 | 4.42 | 3.62 | 3.89 | 3.40 | 3.42 |
| 23 | 6.30 | 6.49 | 5.28 | 4.83 | 4.87 | 4.52 | 3.78 | 4.82 | 2.90 | 2.79 |
| 24 | 6.14 | 6.66 | 5.55 | 5.01 | 4.63 | 4.43 | 3.88 | 4.61 | 3.10 | 3.31 |
| 25 | 6.52 | 6.50 | 5.63 | 5.24 | 4.72 | 4.17 | 4.10 | 4.56 | 3.59 | 3.03 |
| 26 | 6.44 | 5.97 | 5.15 | 5.78 | 4.54 | 4.25 | 3.95 | 4.78 | 2.89 | 3.56 |
| 27 | 6.45 | 6.18 | 5.54 | 5.89 | 4.07 | 4.67 | 4.00 | 4.73 | 2.33 | 3.55 |
| 28 | 5.98 | 6.49 | 5.43 | 5.50 | 3.82 | 4.80 | 3.64 | 4.75 | 2.74 | 3.52 |
| 29 | 6.03 | 6.52 | 5.21 | 5.02 | 4.50 | 4.61 | 3.58 | 4.40 | 2.34 | 3.59 |
| 30 | 6.01 | 6.52 | 5.31 | 5.34 | 4.91 | 4.86 | 3.79 | 4.43 | 2.51 | 3.74 |
| 31 | 5.81 | 6.70 | 5.14 | 5.68 | 4.85 | 5.12 | 3.60 | 4.74 | 2.63 | 2.86 |
| 32 | 6.04 | 6.35 | 5.35 | 5.52 | 5.12 | 4.84 | 3.93 | 4.66 | 2.41 | 3.22 |
| 33 | 6.39 | 6.04 | 5.11 | 5.35 | 5.23 | 4.81 | 4.33 | 4.22 | 2.35 | 3.40 |
| 34 | 6.37 | 6.08 | 5.35 | 5.36 | 4.80 | 5.11 | 3.69 | 4.50 | 2.34 | 3.74 |
| 35 | 6.40 | 6.55 | 5.25 | 5.48 | 4.38 | 4.62 | 4.42 | 4.30 | 3.32 | 3.68 |
| 36 | 6.45 | 6.70 | 5.12 | 5.08 | 4.73 | 4.80 | 4.09 | 4.31 | 3.70 | 3.20 |
| 37 | 6.61 | 6.78 | 5.07 | 5.38 | 4.80 | 4.91 | 3.55 | 4.50 | 3.20 | 3.48 |
| 38 | 6.25 | 6.87 | 5.38 | 5.83 | 4.43 | 4.90 | 3.79 | 4.44 | 3.32 | 2.62 |
| 39 | 6.27 | 6.65 | 5.52 | 5.80 | 4.44 | 5.11 | 3.67 | 4.46 | 3.05 | 2.71 |
| 40 | 6.44 | 6.09 | 5.21 | 5.82 | 4.28 | 4.24 | 4.09 | 4.48 | 2.96 | 3.19 |
| 41 | 6.15 | 6.05 | 4.96 | 5.97 | 3.78 | 4.79 | 4.25 | 4.52 | 2.59 | 3.10 |
| 42 | 6.24 | 6.49 | 5.25 | 5.61 | 3.53 | 4.46 | 4.33 | 4.75 | 2.60 | 3.15 |
| 43 | 6.18 | 6.68 | 5.16 | 5.27 | 3.78 | 4.11 | 3.97 | 4.12 | 2.54 | 2.99 |
| 44 | 6.46 | 6.55 | 5.07 | 5.00 | 4.72 | 4.31 | 3.47 | 4.26 | 2.69 | 2.94 |
| 45 | 5.97 | 6.67 | 5.36 | 5.51 | 4.86 | 5.11 | 3.66 | 4.40 | 2.89 | 3.17 |
| 46 | 6.37 | 6.35 | 5.41 | 5.33 | 4.76 | 4.86 | 3.64 | 4.84 | 3.40 | 3.21 |
| 47 | 6.01 | 5.92 | 5.14 | 5.30 | 4.77 | 4.97 | 3.91 | 4.82 | 3.68 | 3.60 |
| 48 | 6.57 | 6.27 | 5.17 | 5.53 | 4.90 | 5.07 | 4.05 | 4.44 | 2.75 | 3.78 |
| 49 | 6.63 | 6.48 | 5.46 | 5.73 | 4.44 | 4.46 | 3.81 | 4.26 | 3.32 | 3.37 |
| 50 | 6.74 | 6.50 | 5.32 | 5.18 | 4.18 | 4.84 | 4.61 | 4.24 | 3.35 | 2.92 |
| 51 | 6.76 | 6.67 | 5.27 | 5.11 | 4.53 | 4.58 | 3.34 | 5.00 | 3.10 | 3.00 |
| 52 | 6.51 | 6.87 | 5.36 | 5.32 | 4.33 | 4.90 | 3.75 | 4.72 | 2.95 | 2.95 |
| 53 | 6.49 | 6.60 | 5.44 | 5.84 | 4.24 | 4.30 | 3.83 | 4.57 | 2.87 | 3.45 |
| 54 | 6.47 | 6.17 | 5.10 | 5.99 | 4.04 | 4.20 | 4.32 | 4.80 | 2.73 | 2.93 |
| 55 | 6.52 | 5.88 | 5.12 | 5.85 | 3.84 | 4.69 | 4.04 | 4.63 | 3.04 | 3.24 |
| 56 | 6.36 | 6.51 | 4.98 | 5.75 | 3.40 | 4.57 | 4.04 | 4.75 | 2.82 | 3.02 |
| 57 | 6.39 | 6.57 | 4.81 | 5.42 | 3.40 | 5.12 | 4.11 | 4.71 | 3.25 | 3.05 |
| 58 | 6.48 | 6.55 | 5.12 | 4.91 | 4.05 | 4.76 | 3.65 | 4.50 | 2.83 | 3.14 |
| 59 | 6.36 | 6.79 | 5.11 | 5.12 | 3.98 | 4.59 | 3.42 | 4.28 | 2.87 | 2.94 |
| 60 | 5.95 | 6.74 | 5.42 | 5.51 | 5.06 | 4.61 | 3.26 | 4.60 | 3.18 | 2.93 |
| 61 | 6.52 | 6.27 | 5.60 | 4.70 | 4.72 | 4.56 | 3.83 | 4.40 | 2.82 | 2.85 |
| 62 | 6.74 | 6.84 | 5.59 | 4.75 | 4.51 | 4.85 | 3.51 | 4.20 | 3.06 | 3.53 |
| 63 | 6.97 | 6.69 | 5.31 | 4.95 | 4.23 | 4.49 | 3.84 | 4.24 | 3.07 | 3.91 |
| 64 | 6.76 | 6.47 | 5.57 | 4.99 | 4.45 | 4.86 | 3.53 | 3.93 | 2.94 | 3.85 |
| 65 | 6.72 | 6.78 | 5.67 | 5.04 | 4.26 | 4.88 | 3.54 | 3.71 | 3.00 | 3.83 |
| 66 | 6.83 | 6.88 | 5.28 | 5.85 | 4.12 | 4.72 | 3.40 | 4.04 | 3.01 | 3.53 |
| 67 | 6.84 | 6.31 | 5.06 | 5.50 | 3.91 | 4.38 | 3.99 | 4.07 | 2.81 | 3.18 |
| 68 | 6.55 | 6.19 | 5.11 | 5.59 | 3.68 | 4.51 | 4.43 | 4.79 | 2.95 | 3.04 |
| 69 | 6.46 | 6.70 | 5.25 | 5.62 | 3.32 | 4.31 | 4.14 | 4.57 | 2.96 | 3.18 |
| 70 | 6.65 | 6.79 | 5.04 | 5.49 | 3.48 | 4.24 | 4.24 | 4.41 | 2.89 | 2.62 |
| 71 | 6.64 | 6.58 | 5.01 | 5.22 | 3.80 | 4.63 | 3.34 | 4.20 | 2.57 | 2.94 |
| 72 | 6.24 | 6.65 | 5.35 | 4.98 | 4.68 | 4.34 | 3.04 | 4.07 | 2.58 | 3.03 |
| 73 | 6.30 | 7.01 | 5.21 | 5.40 | 4.75 | 4.34 | 3.40 | 4.50 | 2.81 | 3.11 |
| 74 | 6.50 | 6.26 | 4.88 | 5.25 | 4.55 | 4.60 | 3.17 | 4.40 | 2.88 | 3.15 |
| 75 | 6.57 | 6.21 | 5.11 | 4.90 | 4.78 | 4.61 | 3.41 | 4.46 | 2.76 | 3.49 |
| 76 | 6.83 | 6.62 | 5.37 | 5.21 | 4.63 | 4.33 | 3.62 | 3.85 | 2.76 | 2.87 |
| 77 | 6.95 | 6.76 | 5.32 | 5.26 | 4.44 | 4.82 | 3.54 | 4.06 | 3.07 | 3.46 |
| 78 | 6.83 | 6.29 | 5.33 | 5.12 | 4.42 | 4.69 | 3.70 | 4.10 | 2.88 | 3.61 |
| 79 | 6.69 | 6.33 | 5.31 | 5.13 | 4.66 | 4.50 | 3.47 | 3.82 | 3.02 | 3.82 |
| 80 | 6.76 | 6.80 | 5.84 | 5.64 | 4.91 | 4.40 | 3.46 | 3.89 | 2.58 | 3.64 |
| 81 | 6.74 | 6.54 | 5.19 | 5.42 | 4.58 | 4.48 | 3.95 | 3.88 | 2.55 | 3.53 |
| 82 | 6.59 | 6.37 | 4.87 | 5.56 | 4.22 | 4.26 | 4.37 | 4.12 | 3.42 | 3.17 |
| 83 | 6.53 | 6.29 | 4.91 | 5.94 | 3.77 | 4.67 | 4.23 | 4.40 | 2.56 | 2.94 |
| 84 | 6.72 | 6.74 | 5.07 | 5.99 | 3.38 | 4.38 | 4.45 | 4.30 | 3.01 | 2.92 |
| 85 | 6.76 | 6.72 | 4.69 | 5.61 | 3.57 | 4.21 | 3.88 | 4.38 | 2.87 | 3.12 |
| 86 | 6.38 | 6.49 | 4.73 | 5.19 | 4.00 | 4.12 | 3.86 | 4.09 | 2.61 | 2.85 |
| 87 | 6.38 | 6.57 | 5.24 | 5.38 | 4.67 | 4.20 | 3.54 | 3.71 | 2.71 | 3.28 |
| 88 | 6.48 | 6.12 | 5.32 | 5.11 | 4.85 | 4.16 | 3.56 | 3.74 | 2.64 | 2.88 |
| 89 | 6.65 | 6.31 | 4.95 | 5.21 | 4.20 | 4.05 | 3.59 | 3.96 | 3.02 | 2.73 |
| 90 | 6.62 | 6.23 | 5.06 | 5.19 | 4.50 | 4.41 | 3.54 | 4.11 | 3.07 | 2.87 |
| 91 | 6.68 | 6.61 | 6.02 | 5.30 | 4.21 | 4.84 | 3.08 | 3.74 | 3.54 | 3.14 |
| 92 | 6.50 | 6.34 | 5.62 | 5.76 | 4.07 | 4.56 | 3.06 | 3.88 | 3.39 | 3.28 |
| 93 | 6.38 | 6.34 | 5.47 | 5.33 | 4.42 | 4.58 | 3.72 | 3.91 | 3.25 | 3.41 |
| 94 | 6.54 | 6.36 | 5.47 | 5.81 | 4.74 | 4.47 | 3.72 | 3.80 | 2.67 | 3.76 |
| 95 | 6.60 | 6.31 | 5.35 | 5.55 | 4.66 | 4.53 | 3.99 | 4.20 | 3.05 | 3.51 |
| 96 | 6.69 | 6.30 | 4.88 | 5.23 | 4.46 | 4.43 | 4.36 | 4.24 | 3.13 | 4.11 |
| 97 | 6.59 | 6.55 | 4.79 | 5.50 | 4.57 | 4.36 | 4.25 | 3.98 | 3.11 | 3.59 |
| 98 | 6.69 | 6.43 | 4.59 | 5.57 | 4.05 | 4.39 | 3.68 | 4.04 | 2.59 | 3.43 |
| 99 | 6.55 | 6.32 | 4.61 | 5.81 | 3.54 | 4.57 | 3.72 | 4.63 | 2.62 | 2.91 |
| 100 | 6.19 | 6.48 | 4.62 | 5.55 | 4.00 | 3.92 | 3.55 | 4.56 | 2.98 | 3.20 |
| 101 | 6.08 | 6.27 | 4.53 | 5.48 | 5.06 | 3.92 | 3.14 | 4.06 | 3.14 | 3.09 |
| 102 | 6.54 | 6.30 | 5.18 | 5.57 | 4.35 | 4.00 | 3.45 | 4.34 | 3.04 | 2.59 |
| 103 | 6.72 | 6.22 | 5.05 | 4.97 | 4.12 | 4.09 | 3.44 | 4.45 | 3.05 | 2.31 |
| 104 | 6.68 | 6.38 | 5.09 | 5.11 | 4.14 | 4.39 | 3.26 | 3.92 | 3.48 | 2.64 |
| 105 | 6.65 | 6.57 | 5.37 | 5.48 | 3.95 | 4.33 | 3.07 | 3.62 | 3.39 | 2.62 |
| 106 | 6.36 | 6.36 | 5.75 | 6.17 | 4.35 | 4.37 | 3.32 | 3.93 | 3.13 | 3.94 |
| 107 | 6.42 | 5.99 | 5.59 | 5.58 | 4.88 | 4.26 | 4.01 | 3.55 | 3.44 | 3.53 |
| 108 | 6.37 | 6.10 | 5.69 | 5.99 | 5.64 | 4.14 | 4.33 | 3.54 | 3.35 | 3.59 |
| 109 | 6.15 | 5.95 | 5.23 | 5.36 | 4.99 | 4.37 | 4.21 | 4.12 | 3.35 | 3.75 |
| 110 | 6.61 | 6.02 | 4.74 | 5.13 | 4.79 | 4.60 | 3.76 | 3.86 | 3.04 | 3.73 |
| 111 | 6.37 | 6.41 | 4.85 | 5.38 | 4.58 | 4.57 | 3.65 | 3.84 | 2.71 | 3.63 |
| 112 | 6.29 | 6.31 | 5.37 | 5.54 | 4.62 | 4.40 | 3.90 | 4.13 | 2.66 | 3.46 |
| 113 | 6.07 | 6.50 | 4.82 | 5.92 | 4.48 | 4.45 | 3.82 | 4.24 | 2.99 | 4.20 |
| 114 | 6.14 | 6.06 | 4.95 | 5.57 | 4.92 | 4.07 | 3.72 | 4.05 | 3.09 | 3.67 |
| 115 | 6.07 | 5.85 | 5.25 | 5.59 | 5.19 | 3.98 | 4.06 | 4.18 | 3.47 | 3.17 |
| 116 | 6.09 | 6.17 | 5.40 | 5.10 | 5.19 | 4.19 | 3.87 | 4.36 | 3.60 | 3.41 |
| 117 | 6.28 | 6.05 | 5.27 | 4.81 | 4.55 | 3.96 | 3.36 | 4.15 | 3.90 | 2.92 |
| 118 | 6.70 | 6.50 | 5.22 | 5.22 | 4.54 | 3.98 | 3.18 | 3.51 | 3.94 | 3.20 |
| 119 | 6.54 | 6.66 | 6.27 | 5.69 | 4.54 | 4.05 | 3.07 | 3.76 | 4.05 | 3.05 |
| 120 | 6.42 | 6.50 | 5.82 | 5.66 | 4.22 | 4.60 | 2.98 | 3.87 | 3.61 | 3.73 |
| 121 | 6.26 | 6.10 | 6.04 | 5.53 | 4.65 | 4.96 | 3.51 | 3.91 | 2.96 | 3.50 |
| 122 | 6.21 | 6.10 | 5.76 | 5.43 | 4.94 | 4.58 | 4.11 | 3.72 | 3.05 | 3.54 |
| 123 | 6.23 | 6.01 | 5.82 | 4.96 | 5.13 | 4.32 | 4.29 | 3.77 | 3.11 | 3.19 |
| 124 | 6.23 | 5.99 | 5.62 | 5.22 | 5.08 | 3.99 | 4.09 | 4.21 | 2.96 | 3.51 |
| 125 | 6.41 | 5.98 | 5.44 | 5.02 | 4.96 | 4.27 | 3.76 | 4.17 | 3.30 | 3.64 |
| 126 | 6.46 | 6.47 | 5.22 | 5.21 | 5.08 | 4.28 | 3.43 | 4.09 | 2.87 | 3.69 |
| 127 | 6.05 | 6.64 | 5.38 | 4.74 | 4.79 | 4.27 | 3.51 | 4.42 | 3.34 | 3.91 |
| 128 | 6.11 | 6.38 | 5.06 | 5.69 | 4.60 | 4.38 | 3.16 | 4.27 | 3.95 | 4.02 |
| 129 | 6.10 | 6.28 | 5.42 | 5.37 | 4.79 | 4.35 | 3.46 | 4.22 | 3.39 | 3.54 |
| 130 | 6.03 | 6.41 | 5.86 | 5.22 | 4.66 | 4.13 | 4.12 | 4.22 | 3.87 | 3.13 |
| 131 | 6.23 | 6.20 | 5.77 | 5.06 | 4.39 | 4.24 | 3.26 | 4.43 | 3.83 | 2.50 |
| 132 | 6.13 | 6.03 | 5.66 | 5.07 | 4.43 | 4.24 | 3.47 | 3.81 | 3.43 | 2.68 |
| 133 | 6.50 | 6.43 | 5.82 | 5.18 | 4.75 | 4.40 | 3.59 | 3.67 | 3.76 | 2.93 |
| 134 | 6.30 | 6.57 | 5.49 | 5.40 | 4.60 | 4.48 | 2.84 | 3.85 | 3.09 | 3.25 |
| 135 | 6.29 | 6.58 | 6.00 | 5.53 | 4.07 | 4.85 | 2.81 | 4.01 | 2.96 | 3.64 |
| min | 5.63 | 5.85 | 4.53 | 4.70 | 3.32 | 3.79 | 2.81 | 3.51 | 1.98 | 2.31 |
| max | 6.97 | 7.01 | 6.27 | 6.17 | 5.64 | 5.12 | 4.61 | 5.00 | 4.05 | 4.20 |
| mean | 6.37 | 6.40 | 5.32 | 5.38 | 4.51 | 4.48 | 3.71 | 4.22 | 3.01 | 3.30 |
| s.d. | 0.28 | 0.25 | 0.33 | 0.31 | 0.47 | 0.31 | 0.39 | 0.33 | 0.40 | 0.37 |

**Supplementary Discussion**

The effective elastic properties of polycrystalline materials are generally evaluated using the Voigt, Reuss, and Hill averages, each relying on distinct assumptions about how strain and stress are distributed among the constituent grains. The Voigt average assumes a uniform strain across all crystallites. The macroscopic stiffness tensor under this assumption is obtained by directly averaging the single-crystal stiffness tensors. This neglects stress heterogeneity and typically yields an upper bound for the elastic moduli. In contrast, the Reuss average assumes a uniform stress throughout the whole system. The effective elastic response is derived by averaging the compliance tensors of individual grains and then inverting the result to obtain stiffness. For an anisotropic orthorhombic system, the independent elastic stiffness are nine: *C*_11_, *C*_22_, *C*_33_, *C*_23_, *C*_31_, *C*_12_, *C*_44_, *C*_55_, and *C*_66_. The Voigt average is expressed in terms of elastic stiffness (*C*_ij_) as follows.

$B_{V}=\frac{1}{9}\left\{C_{11}+C_{22}+C_{33}+2(C_{12}+C_{23}+C_{13})\right\}$,
$G_{V}=\frac{1}{15}\left\{C_{11}+C_{22}+C_{33}-C_{12}-C_{23}-C_{13}+3(C_{44}+C_{55}+C_{66})\right\}$.

Similarly, Reuss averages are expressed using elastic compliance (S_ij_), which is the reciprocal of elastic stiffness:

$B_{R}=\frac{1}{S_{11}+S_{22}+S_{33}+2(S_{12}+S_{23}+S_{13})}$,

$G_{R}=\frac{15}{4(S_{11}+S_{22}+S_{33})-4(S_{12}+S_{23}+S_{13})+3(S_{44}+S_{55}+S_{66})}$.

Considering cubic symmetry, the averages are given as follows:

$B_{V}=B_{R}=\frac{C_{11}+2C_{12}}{3}$,
$G_{V}=\frac{C_{11}-C_{12}+3C_{44}}{5}$, $G_{R}=\frac{5(C_{11}-C_{12})C_{44}}{4C_{44}+3(C_{11}-C_{12})}$.

This method generally provides a lower bound for the elastic moduli. The Hill average is widely adopted since real polycrystalline materials exhibit behavior between these two limiting cases, which are given by taking the arithmetic mean of the Voigt and Reuss averages.

$$B_{VRH}=\frac{K_{V}+K_{R}}{2},$$

$$G_{VRH}=\frac{G_{V}+G_{R}}{2}$$

**Supplementary References**

1. Voigt, W. Lehrbuch der Kristallphysik. Leipzig. Teubner,1928.
2. Reuss A. Berechnung der Fließgrenze von Mischkristallen auf Grund der Plastizitätsbedingung für Einkristalle. Zeitschrift für Angewandte Mathematik und Mechanik. 1929;**9:**49–58.
3. Hill R. The Elastic Behaviour of a Crystalline Aggregate. Proc Phys Soc Sect A. 1952;**65**:349–354.
